# Biodegradation of polyethylene terephthalate microplastics by *Paenibacillus naphthalenovorans* PETKKU2: Response surface optimization and genomic evidence for an alternative degradation mechanism

**DOI:** 10.1371/journal.pone.0341623

**Published:** 2026-02-04

**Authors:** Aophat Choonut, Nantharat Wongfaed, Auraiwan Poolpol, Sophon Boonlue, Kitirote Wantala, Onruthai Pinyakong, Pensri Plangklang, Alissara Reungsang

**Affiliations:** 1 Department of Biotechnology, Faculty of Technology, Khon Kaen University, Khon Kaen, Thailand; 2 Research Group for Development of Microbial Hydrogen Production Process from Biomass, Khon Kaen University, Khon Kaen, Thailand; 3 Department of Microbiology, Faculty of Science, Khon Kaen University, Khon Kaen, Thailand; 4 Department of Chemical Engineering, Faculty of Engineering, Khon Kaen University, Khon Kaen, Thailand; 5 Department of Microbiology, Faculty of Science, Chulalongkorn University, Bangkok, Thailand; 6 Academy of Science, Royal Society of Thailand, Bangkok, Thailand; Mahatma Jyotiba Phule Rohilkhand University, INDIA

## Abstract

This study establishes *Paenibacillus naphthalenovorans* PETKKU2, isolated from landfill soil in Thailand, as the first reported member of this species capable of degrading polyethylene terephthalate microplastics (PET-MP). Initial screening identified PETKKU2 as the most efficient degrader among ten isolates, achieving 6.07 ± 0.18% weight loss after 35 days at 37°C. Response surface methodology optimization of pH, nitrogen concentration, and PET-MP loading enhanced degradation to 9.48 ± 0.21%, closely matching the predicted maximum of 11.15% and representing 96% improvement over baseline conditions. Integrated analytical characterization (FTIR, SEM, GC-MS) revealed an alternative degradation mechanism distinct from classical PETase-MHETase pathways. FTIR analysis confirmed extensive polymer oxidation with 41% reduction in ester carbonyl groups, while SEM demonstrated progressive surface erosion. Critically, the absence of mono(2-hydroxyethyl) terephthalate (MHET) intermediates, combined with whole-genome analysis revealing thermostable lipases, carboxylesterases, and dioxygenases, but no PETase/MHETase homologs, indicates novel enzymatic routes. Operating under mesophilic conditions (37 °C), PETKKU2 eliminates energy-intensive heating requirements while achieving performance comparable to established thermophilic degraders. These findings establish a promising platform for sustainable PET-MP bioremediation and advance understanding of alternative microbial plastic degradation mechanisms.

## Introduction

Society faces an escalating threat from plastic accumulation, akin to the risks posed by greenhouse gas emissions and land degradation [1]. Plastic waste in landfills and natural environments is expected to reach 12 billion tons by 2050 if left unchecked [2]. Polyethylene terephthalate (PET), a common thermoplastic polymer found in products like fibers, bottles, and filaments, plays a significant role in daily life while contributing substantially to environmental contamination.

The environmental persistence of PET creates cascading ecological problems. Its slow degradation rate leads to long-term accumulation in ecosystems, while thermal breakdown of PET-containing materials can release toxins and heavy metals such as cadmium and lead [3]. When exposed to ultraviolet light, water, and heat, PET can fragment into microplastics (MP, PET-MP) [4], which readily absorb toxic chemicals and pose serious health risks to organisms throughout the food chain [5,6]. Conventional disposal methods like incineration and landfilling generate secondary pollutants, making them unsustainable long-term solutions for addressing PET-MP contamination [7,8].

Microbial biodegradation offers a sustainable alternative to conventional waste management, utilizing natural processes to reduce waste and minimize pollution [9]. The foundation for enzymatic plastic degradation was established in 1977 with the identification of lipases and esterases capable of degrading polyesters, including PET [10]. More recent studies have further characterized these enzymes and their PET-degrading activity [11,12].

A major breakthrough in enzymatic PET degradation occurred with the identification of a cutinase from *Thermobifida fusca* that efficiently hydrolyzed PET, as first reported by Müller et al. in 2005 [13]. Subsequently, in 2016, *Ideonella sakaiensis* 201-F6 was isolated, which degrades PET through specialized enzymes, polyethylene terephthalate hydrolase (PETase) and mono(2-hydroxyethyl) terephthalate hydrolase (MHETase) [14]. While the discovery of *I. sakaiensis* greatly accelerated research in the field, importantly, cutinases such as those from *T. fusca* generally exhibit higher PET-degrading efficiency, even compared to engineered PETase variants.

Moreover, the present study emphasizes the advantages of mesophilic enzymes derived from naturally occurring organisms, which operate effectively under mild environmental conditions without requiring protein engineering or elevated temperatures. This highlights the industrial and ecological relevance of the PETKKU2 strain as a sustainable biocatalyst for PET degradation. Generally, thermophilic enzymes are highly stable and show strong catalytic activity at elevated temperatures, but their activities markedly decrease at moderate temperatures compared to their mesophilic counterparts. In contrast, mesophilic enzymes, though less thermally stable, often exhibit greater catalytic efficiency under mild conditions, reflecting the well-documented inverse correlation between enzyme stability and activity [15,16]. This discovery has since catalyzed advancements in synthetic biology, enabling the enhancement of enzymes and microbial strains to improve PET biodegradation efficiency [9].

Building on these foundations, numerous microorganisms have demonstrated PET and PET-MP degradation capabilities, including *Microsphaeropsis arundinis* [17], *I. sakaiensis* [18], *Pseudomonas marincola* WJ1 [19], *Streptococcus pyogenes* [20], *Pseudomonas* sp. [21], *Bacillus cereus* [22], *Priestia aryabhattai* VT 3.12 [23], *Bacillus* sp. BCBT21 [24], *Spirulina* sp. [25], as well as *Aspergillus* sp. and *Vibrio* sp. [26]. Recently, a thermostable PETase from *Kibdelosporangium aridum* was discovered, demonstrating high hydrolytic activity toward PET at elevated temperatures [27]. This finding expands the diversity of microbial PET-degrading enzymes and provides a useful comparison for understanding the metabolic potential of *Paenibacillus naphthalenovorans* PETKKU2 in PET degradation. However, the degradation efficiencies of these organisms vary significantly with environmental conditions, and many exhibit limitations in practical applications, highlighting the critical need to identify new microbial strains with enhanced biodegradation capabilities and increased resistance to environmental stresses.

Despite these advances, the biodegradation efficiencies of identified PET-degrading strains are often constrained by non-optimal environmental conditions. Recent studies on polymer and plastic biodegradation demonstrates that factors such as pH, nitrogen availability, and plastic concentration significantly influence microbial degradation performance. pH strongly influences polyester hydrolysis, affecting polymer chain cleavage and enzyme stability [28]. Nitrogen availability can enhance hydrolytic enzyme expression and microbial metabolism [29], while substrate concentration can induce enzyme production without causing inhibitory effects, consistent with observations on biofilm formation and microbial activity on microplastics [30].

However, systematic optimization approaches, particularly response surface methodology (RSM), remain underutilized in PET-MP biodegradation research. RSM provides a robust statistical framework for evaluating and modeling the effects of multiple environmental variables, enabling the identification of optimal conditions that maximize degradation efficiency. Incorporating such optimization strategies could unlock hidden metabolic potential in promising bacterial strains and significantly enhance their practical applicability in real-world bioremediation systems.

To address these gaps, this study isolated PET-MP degrading bacteria from landfill soil, optimized their performance through systematic environmental manipulation, and characterized their degradation mechanisms using integrated analytical and genomic approaches. The results provide insights into alternative degradation pathways and establish a foundation for sustainable microplastic bioremediation.

## Materials and methods

### Microplastic preparation

Commercial-grade PET pellets were purchased from Petro Plus Chemical Co., Ltd., Bangkok, Thailand. The pellets were white, opaque, and rod-shaped with an average length of approximately 5 mm and a width of about 2.5 mm. To simulate environmentally relevant microplastic morphology and increase surface area for biodegradation experiments, the PET pellets were ground using a freezer mill (NSTDA, NCTC) and sieved (C-Tech-TPS02–0005, Siam Intercorp, Thailand) to obtain irregular fragments between 1 and 5 mm.

Following a modified protocol from Jeon et al. (2021), particles were washed with 70% ethanol for 45 minutes, dried at 50 °C for 24 hours, and UV-sterilized (254 nm, 15 minutes). Gravimetric analysis confirmed no significant weight loss during preparation, establishing a reliable baseline for biodegradation measurements. Additionally, FTIR analysis of the prepared PET-MP (S1 Fig) showed characteristic PET peaks without additional oxidation bands, confirming that the UV sterilization and washing procedures did not measurably alter PET composition or introduce significant surface modifications. Therefore, these treatments are unlikely to have affected subsequent biodegradation results.

### Culture media and chemicals

Four media were used for microbial screening, isolation, and characterization. Mineral salt (MS) medium for screening contained (g/L): 9.0 Na_2_HPO_4_⋅12H₂O, 1.5 KH_2_PO_4_, 0.1 NH_4_Cl, and 0.2 MgSO_4_ ⋅ 7H_2_O [31], enabling selection of microorganisms using PET-MP as sole carbon source. Nutrient broth (NB) medium for inoculum preparation contained (g/L): 5.0 peptone and 3.0 yeast extract. Nutrient agar (NA) medium for isolation added 15.0 g/L agar to NB [32]. Butter agar (BA) medium for lipase detection contained (g/L): 5.0 peptone, 2.5 yeast extract, 15.0 agar, 0.01 methylene blue, 25 mL/L liquid butter, and 5 mL/L Tween 80 [33]. All chemicals were analytical grade from Merck (Germany) and HiMedia (India).

### Collection and characterization of soil samples

Soil samples were collected from plastic disposal areas at a landfill in Khon Kaen, Thailand. Samples were obtained from six locations at depths up to 25 cm to target microbial communities adapted to plastic degradation. No specific permits were required for sample collection, as the study did not involve endangered species or restricted areas, and access to the site was granted by the landfill management. Samples were transferred to sterile 100 mL bottles, transported in an ice box, and stored at −20 °C until processing.

Environmental parameters were measured on-site using a pH meter and thermometer. The predominantly black loam soil exhibited heterogeneous conditions across sampling points (pH 5–10, varying moisture content and nutrient availability), with temperatures ranging from 29–35 °C, enabling the collection of microorganisms adapted to varying environmental factors. Detailed sampling information, including GPS coordinates, pH, temperature, and physical characteristics, is provided in S1 Table.

### Enrichment and screening of PET-MP-degrading microorganisms

**Fig 1:**
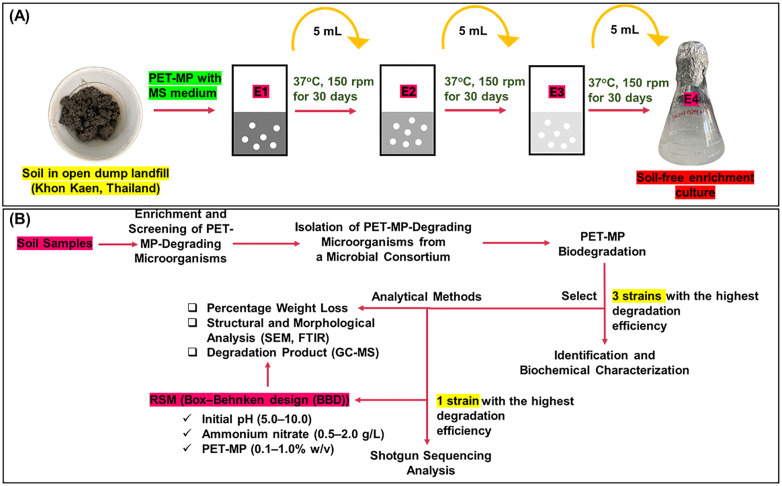
Sequential enrichment and experimental workflow for isolating polyethylene terephthalate microplastics (PET-MP)-degrading bacteria from landfill soil. **(A)** Sequential enrichment process (E1–E4) for isolating PET-MP-degrading consortia from landfill soil; **(B)** Experimental workflow diagram showing the isolation, characterization, and analysis of PET-MP-degrading bacteria.

A sequential enrichment strategy was used to isolate PET-MP-degrading microorganisms, based on the principle that successive transfers selectively amplify organisms with specific degradation capabilities [34]. For each enrichment cycle, 5 g of soil was inoculated into 50 mL of MS medium containing 1% (w/v) PET-MP as the sole carbon source and incubated at 37 °C with agitation at 150 rpm for 30 days (E1). Subsequently, 5 mL aliquots were transferred to fresh medium and incubated under identical conditions. This process was repeated for four complete cycles (E1–E4) to obtain soil-free consortia (**Fig 1A**).

The resulting consortia were first evaluated for lipase production as a preliminary screening indicator of potential esterase activity relevant to polyester degradation, and consortia showing lipase activity were subsequently tested for actual PET-MP degradation capability before isolation of individual strains. Among the six consortia (CPET-KKU1–CPET-KKU6), three (CPET-KKU3, CPET-KKU4, and CPET-KKU6) exhibited positive lipase activity and were selected for further analysis. From these three consortia, a total of ten bacterial isolates were obtained and designated PETKKU1–PETKKU10. These isolates underwent lipase activity testing, 16S rRNA gene sequencing, and biochemical characterization. Promising strains were subjected to biodegradation testing and post-degradation analysis using spectroscopic and microscopic techniques. The most effective strain was selected for whole-genome sequencing to characterize its genetic basis for PET-MP degradation (**Fig 1B**).

### Lipase-producing activities of the enriched PET-MP-degrading microbial consortium

Microbial consortia from the enrichment process were screened for lipase production. For each assay, 0.1 mL of consortium culture in logarithmic phase (OD600 = 1.0) was inoculated onto BA medium and incubated at 37 °C for 48 hours [35]. Lipase production was quantified by measuring the clear zone diameter around colonies using a micrometer, with the clear zone index calculated as:


$$\text{Clear}\;\text{zone}\;\text{index}=\;\frac{\text{Clear}\;\text{zone}\;\text{diameter}\;}{\text{Colony}\;\text{diameter}}$$ (1)

This screening method was based on established correlations between lipase production and plastic degradation capability, as lipases hydrolyze ester bonds in PET polymers [36]. Consortia showing significant lipase production were selected for purification into individual strains, which were then reassessed for lipase activity to identify candidates with the highest potential for PET-MP biodegradation.

### Isolation and purification of lipase-producing microorganisms from PET-MP-degrading consortia

Bacterial strains were isolated from lipase-positive consortia through serial dilution and plating on NA medium. After incubation at 37 °C for 48 hours, morphologically distinct colonies were selected and purified through successive streak-plating until homogeneous colonies were obtained. Each purified colony was cultivated in 5 mL NB medium (37 °C, 150 rpm, 24 hours), then transferred to 25 mL fresh NB medium and incubated until reaching logarithmic phase (OD600 = 1.0).

Each isolated strain was reassessed for lipase production using the clear zone method to ensure maintenance of lipolytic activity in monoculture conditions, as enzymatic profiles can differ between consortia and individual isolates. This reassessment was critical for selecting the most promising candidates for biodegradation testing. Lipase-positive strains were preserved in 10% glycerol at −20 °C for long-term storage.

### PET-MP biodegradation by isolated microorganisms

Lipase-producing bacterial isolates were tested for PET-MP biodegradation capability. A 10% (v/v) inoculum of each strain in logarithmic phase was transferred to 50 mL MS medium containing 1% (w/v) PET-MP as the sole carbon source. Cultures were incubated at 37 °C with shaking at 150 rpm for 35 days. Samples were collected every 7 days to monitor microbial growth, measured as optical density at 600 nm (OD600) using a UV-VIS spectrophotometer (MODEL-UV5100, Japan), and pH changes, measured using a calibrated pH meter (pH Series, HORIBA).

After incubation, cultures were filtered to recover the remaining PET-MP particles. Biodegradation efficiency was determined by calculating the percentage weight loss. The three best-performing strains were selected for further analysis. These strains underwent detailed characterization using GC-MS to identify degradation products, FTIR and SEM to analyze structural and morphological changes in the PET-MP. Taxonomic identification was performed through 16S rRNA gene sequencing and biochemical profiling. The most efficient strain was subjected to whole-genome sequencing for genetic characterization of its PET-MP degradation machinery.

### Identification and biochemical characterization of three strains isolated

The three bacterial isolates with highest PET-MP biodegradation efficiency were identified and characterized biochemically. For taxonomic identification, 16S rRNA genes were amplified using a PCR protocol adapted from Wagner et al. (1998) with the OmniPCR kit (Gibthai, Thailand). Genomic DNA from each isolate was extracted using the QIAamp DNA Mini Kit (Qiagen, Hilden, Germany) following the manufacturer’s protocol. DNA quality and concentration were assessed using a NanoDrop ND-1000 spectrophotometer and a Qubit 2.0 Fluorometer (Thermo Fisher Scientific, Waltham, MA, USA).

Each 25 μL reaction contained 10.5 μL distilled water, 12.5 μL OmniPCR master mix, 1 μL of each primer (10 pmol/L), and 50 ng of template DNA. The 16S rRNA gene was amplified using universal bacterial primers 27F (5′-AGAGTTTGATCMTGGCTCAG-3′) and 1492R (5′-TACGGYTACCTTGTTACGACTT-3′) (Macrogen, Seoul, Korea). PCR conditions included initial denaturation at 95°C for 3 minutes, followed by 30 cycles of denaturation (95 °C, 30 seconds), annealing (54 °C, 30 seconds), and extension (72 °C, 1.5 minutes), with final extension at 72 °C for 5 minutes [37]. PCR products were analyzed by gel electrophoresis (70 V, 40 minutes, 1.5% agarose gel in 1 × TAE buffer), visualized under UV light, and sequenced by Ward Medic Co., Ltd. using an Applied Biosystems 3730/3730xl DNA Analyzer. Sequences were analyzed with Sequencing Analysis Software v7.0 and identified using BLAST at NCBI.

Biochemical characterization was performed using the KB013 Hi Bacillus™ identification kit, selected for its suitability for Gram-positive bacteria, particularly *Bacillus* species. This system provides substrate utilization tests to reveal metabolic capabilities relevant to environmental adaptation and biodegradation potential [38,39].

### Experimental design for optimization of PET-MP biodegradation

To enhance PET-MP biodegradation by *P. naphthalenovorans* PETKKU2, RSM was employed using a Box–Behnken design (BBD). Three independent variables, initial pH (5.0–10.0), ammonium nitrate concentration (0.5–2.0 g/L), and PET-MP concentration (0.1–1.0% w/v), were selected based on preliminary studies. The selection of these variables was based on their critical roles in microbial growth, enzymatic activity, and substrate availability. Previous studies on microbial polymer degradation have shown that pH strongly influences polyester hydrolysis, affecting polymer chain cleavage and enzyme stability [28]. Nitrogen availability can enhance hydrolytic enzyme expression and microbial metabolism [29], while substrate concentration can induce enzyme production without causing inhibitory effects, consistent with observations on biofilm formation and microbial activity on microplastics [30].

A total of 17 experimental runs, including five center points, were generated using Design-Expert software (v13, Stat-Ease Inc., USA). Each experimental unit consisted of 50 mL of MS medium in 100 mL Erlenmeyer flasks, inoculated with 10% (v/v) active PETKKU2 culture, and incubated at 37 °C with shaking at 150 rpm for 35 days. PET-MP degradation was quantified as percentage weight loss. A second-order polynomial model was fitted to the experimental data, and the statistical significance of each factor and interaction term was determined using analysis of variance (ANOVA). The optimal conditions predicted by the model were validated in triplicate. This approach enabled the identification of key environmental parameters that significantly influence PET-MP biodegradation efficiency and revealed enhanced degradation performance under optimized conditions.

### Shotgun sequencing analysis

For whole-genome sequencing of strain PETKKU2, genomic DNA was similarly prepared and verified for quality and concentration as described above. Whole-genome sequencing was performed by Novogene (Beijing, China) on the Illumina NovaSeq 6000 platform through U2Bio (Thailand) Co., Ltd. Libraries were prepared using the NEBNext® Ultra™ DNA Library Prep Kit following manufacturer’s protocol. Sequencing was conducted in paired-end mode (PE150), generating 16.4 million paired-end reads (150 + 150 bp) and 1.1 million long reads.

Genomic analysis enabled identification of key genes involved in PET degradation pathways, including hydrolases, lipases, esterases, and enzymes for metabolizing degradation products. The analysis also revealed genes associated with stress tolerance and environmental adaptation that likely contribute to the strain’s effective plastic degradation capabilities. These findings provide insights into the molecular mechanisms of PET-MP biodegradation and potential genetic resources for biotechnological applications in plastic waste remediation.

### Analytical methods and statistical analysis

#### Percentage weight loss analysis.

After the 35-day incubation period, PET-MP particles were recovered by filtration under reduced pressure (vacuum filtration) using Whatman No. 1 filter paper. The washing procedure was modified from Muangchinda and Pinyakong (2024) [40]. Particles were washed five times with 2% sodium dodecyl sulfate (SDS) solution (1 min per wash), followed by three rinses with distilled water and three rinses with 95% ethanol to remove all microbial cells and residues. This stringent washing protocol was specifically applied to samples for gravimetric analysis to ensure that measured weight loss reflects actual polymer degradation rather than biomass removal. Control samples (PET-MP incubated without bacteria) confirmed that the washing protocol did not cause mechanical weight loss, with unwashed control samples showing <0.1% weight variation, validating that measured weight losses primarily reflect biodegradation rather than biomass removal artifacts. Samples were dried at 50 °C for 24 hours, then placed in a desiccator for 6 hours before weighing using a precision analytical balance (Precisa XB 220A 4-balance, Switzerland) [40]. Biodegradation efficiency was calculated as percentage weight loss using Equation 2:


Percentage weight loss =[(Initial weight −Final weight)/Initial weight ×100 (2)

#### Structural and morphological analysis.

PET-MP structural changes were analyzed using FTIR (Tensor II, Bruker, Germany) with a Platinum ATR accessory across 4000–400 cm ⁻ ¹ to detect functional group modifications. Surface morphology was examined by SEM (LEO 1450VP) at 10.0 kV. For SEM, samples were mounted on carbon adhesive tape, demagnetized, and gold-coated to prevent charging during imaging [4]. To visualize progressive stages of biodegradation, SEM samples were subjected to differential washing protocols: gentle washing (single rinse with distilled water) was applied to samples for Fig 7B–7C to preserve biofilm structures and microbial colonization, while the stringent washing protocol (as described for gravimetric analysis) was applied to samples for Fig 7D to visualize polymer surface erosion after biofilm removal. These complementary techniques provided evidence of biodegradation through detection of chemical modifications and visualization of surface erosion patterns, respectively.

#### Degradation product analysis.

Degradation products were analyzed using GC-MS (Agilent Technologies 5977B Inert Plus MSD/GC 8890 with Headspace system). Metabolite analysis was performed on culture supernatant collected at day 35 only, representing endpoint degradation products. Sample preparation involved extracting 3 mL of culture supernatant with 1 mL dichloromethane, followed by concentration to 500 μL. A 1 μL sample was injected into a DB-5MS capillary column [41]. GC-MS conditions included helium carrier gas at 1.2 mL/min flow rate, inlet temperature of 225 °C (Splitless mode), and temperature gradient from 50 °C (3 min hold) to 300 °C at 6°C/min. The transfer line and ion source were maintained at 250 °C, with electron ionization at 70 eV [41]. Compounds were identified by comparing mass spectra with the NIST library and published degradation pathways, providing insights into the biochemical mechanisms of PET-MP biodegradation.

### Statistical analysis

All experiments were performed in triplicate, with data presented as mean values ± standard deviation (SD). Statistical significance was determined using one-way analysis of variance (ANOVA) at a 95% confidence interval (*p* ≤ 0.05), followed by Tukey’s Honestly Significant Difference (HSD) post-hoc test to determine specific differences between groups. Differences were considered statistically significant when *p* ≤ 0.05. All statistical analyses were performed using the Data Analysis Add-in function in Microsoft Excel 2010 with the XLSTAT extension.

## Results and discussion

### Isolation and screening of PET-MP degrading consortium

**Fig 2:**
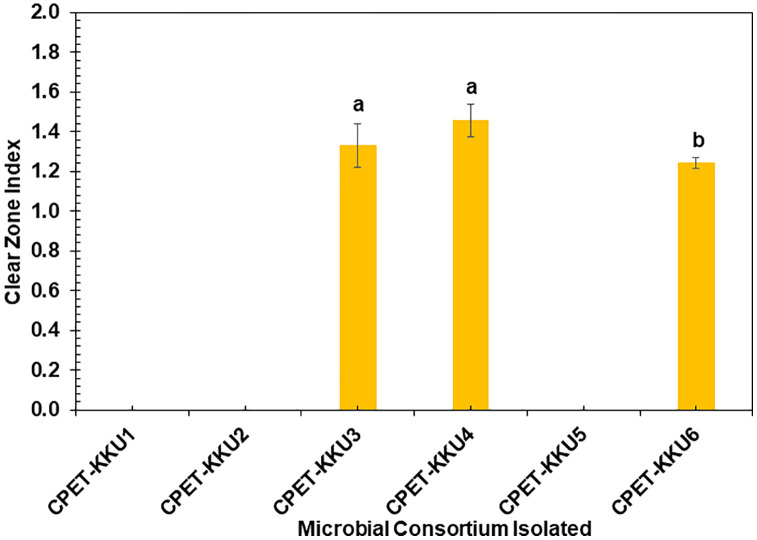
Lipase production capacity of six enriched microbial consortia measured by clear zone index on butter agar medium. Error bars represent standard deviation (n = 3).

The PET-MP-degrading microbial consortia were screened from soil samples at six locations using lipase activity tests. **Fig 2** shows the lipase activity, assessed by the clear zone index on the BA medium. CPET-KKU3, CPET-KKU4, and CPET-KKU6 exhibited clear zones, indicating lipase production, whereas CPET-KKU1, CPET-KKU2, and CPET-KKU5 did not. CPET-KKU4 had the highest clear zone index (1.46 ± 0.08), followed by CPET-KKU3 (1.33 ± 0.10) and CPET-KKU6 (1.24 ± 0.03). These findings suggest that CPET-KKU3, CPET-KKU4, and CPET-KKU6 produce lipase, which may contribute to PET-MP hydrolysis, consistent with previous studies that reported lipase can degrade PET [42].

The role of lipase in plastic degradation is well-established, and its production is widely utilized as a screening method to identify plastic-degrading microorganisms due to its simplicity, rapidity, and efficiency, making it particularly suitable for screening large numbers of samples [35]. Based on these results, consortia CPET-KKU3, CPET-KKU4, and CPET-KKU6 were selected for further isolation of pure strains.

### Isolation and purification of lipase-producing microorganisms from three PET-MP-degrading consortia

A total of ten bacterial isolates were obtained from the three enriched microbial consortia and designated as PETKKU1–PETKKU10, with CPET-KKU3 yielding five isolates (PETKKU1–PETKKU5), CPET-KKU4 yielding three isolates (PETKKU6–PETKKU8), and CPET-KKU6 yielding two isolates (PETKKU9–PETKKU10). All ten isolates were initially screened for PET-MP degradation activity. Based on preliminary degradation assays, three isolates demonstrating the highest degradation efficiency were selected for detailed molecular identification and biochemical characterization: PETKKU2 (from consortium CPET-KKU3), PETKKU6 (from consortium CPET-KKU4), and PETKKU10 (from consortium CPET-KKU6). The remaining seven isolates were not subjected to molecular identification, as the focus was directed toward comprehensive characterization of the most promising PET-degrading strains.

The colonies appeared small and exhibited white, cream, or yellow colors, with lens-shaped or smooth/rough textures, characteristic of Bacillus species. All isolates were Gram-positive, as confirmed by Gram staining (S2 Table). Gram-positive bacteria, particularly Bacillus species and closely related strains, are commonly associated with the degradation of complex organic materials and demonstrate adaptability to various environmental conditions [43,44]. These purified strains were selected for further screening to evaluate their lipase production, which would help assess their specific contributions to the degradation of PET-MP.

### Screening of lipase-producing bacteria from ten isolated strains

**Fig 3:**
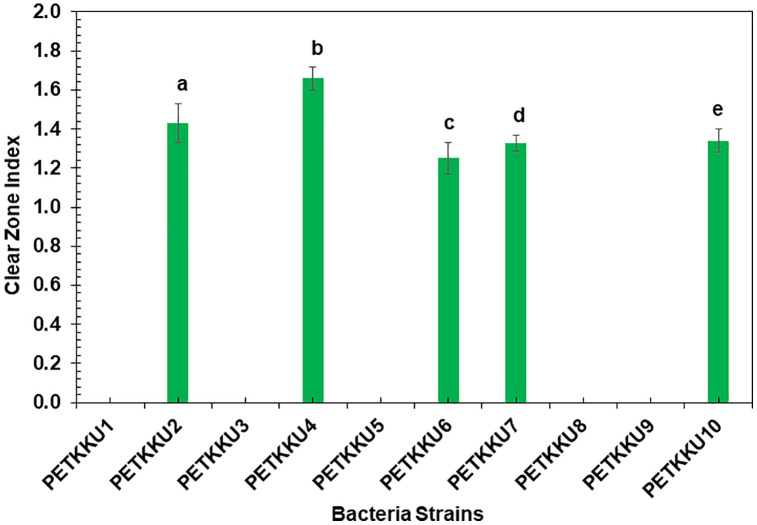
Lipase production capacity of ten bacterial isolates measured by clear zone index on butter agar medium. Error bars represent standard deviation (n = 3).

Lipase production was evaluated in ten bacterial strains cultured on BA medium with liquid butter as the carbon source. Five strains showed significant lipase production, with PETKKU4 having the highest index (1.66 ± 0.06), followed by PETKKU2 (1.43 ± 0.10), PETKKU10 (1.34 ± 0.06), PETKKU7 (1.33 ± 0.04), and PETKKU6 (1.25 ± 0.08) (**Fig 3**), indicating the potential for PET-MP biodegradation. When compared to microbial consortia, CPET-KKU4 had the highest index (1.46 ± 0.08), followed by CPET-KKU3 (1.33 ± 0.10) and CPET-KKU6 (1.24 ± 0.03) (**Fig 2**).

The minimal differences between isolated strains and consortia suggest that individual isolates may produce lipases more efficiently due to specialized metabolic pathways. This supports previous studies highlighting the advantages of monoculture biodegradation, such as easier growth and better control compared to mixed cultures that require more complex management [45]. Based on their strong lipase production, these five strains were selected for further degradation testing.

### PET-MP degradation performance of five selected bacterial strains

**Fig 4:**
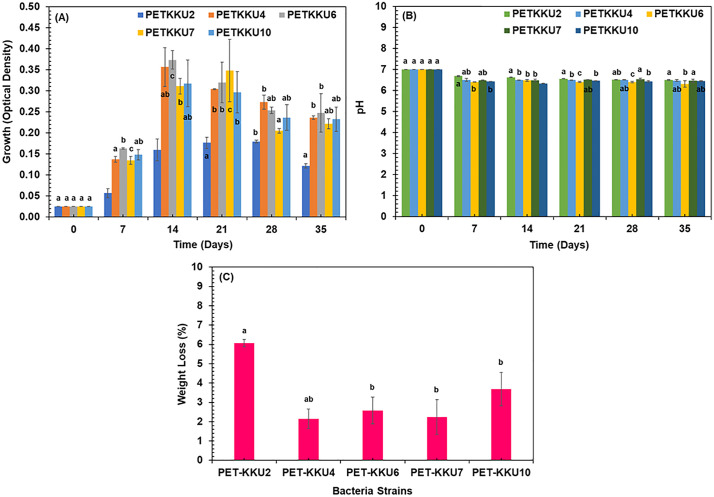
Biodegradation of polyethylene terephthalate microplastics (PET-MP) by five bacterial isolates over 35 days. **(A)** Bacterial growth; (B) pH changes in the culture medium; and **(C)** Final degradation efficiency expressed as percentage weight loss. Error bars represent standard deviations (n = 3). Different letters indicate significant differences among samples at p < 0.05 (Tukey’s test). Bars sharing the same letter are not significantly different.

The biodegradation efficiency of PET-MP by these strains can be influenced by environmental factors, including pH, temperature, nutrient availability, and the presence of inhibitory substances. These conditions critically affect enzyme stability and microbial metabolism by modulating enzyme catalytic activity, microbial growth rates, and metabolic efficiency [46]. The growth of five bacterial strains (PETKKU2, PETKKU4, PETKKU6, PETKKU7, and PETKKU10) was assessed in MS medium containing PET-MP as the sole carbon source. Bacterial growth was monitored by measuring OD₆₀₀ every 7 days over a 35-day incubation period. Among these isolates, PETKKU6 exhibited the most robust growth, reaching a maximum OD₆₀₀ of 0.374 ± 0.022 at day 14. PETKKU4 and PETKKU10 also achieved their maximum OD₆₀₀ around day 14 (0.357 ± 0.046 and 0.318 ± 0.056, respectively), whereas PETKKU7 reached its maximum later, around day 21 (0.311 ± 0.018). PETKKU2 showed the lowest growth, with a maximum OD₆₀₀ of approximately 0.160 ± 0.026 observed at day 14. Statistical analysis (one-way ANOVA followed by Tukey’s HSD test, p < 0.05) indicated that the maximum OD₆₀₀ of PETKKU6 was significantly higher than those of PETKKU2, PETKKU7, and PETKKU10, but not significantly different from PETKKU4 (**Fig 4A**). These differences suggest distinct metabolic capacities among the bacterial strains, potentially influenced by extracellular enzyme production and their ability to degrade PET-MP [47]. The observed growth variation aligns with findings from similar studies [48,49] and underscores the potential of these strains for bioremediation applications.

During the 35-day experiment, gradual pH declines were observed in the culture medium for all strains, decreasing from an initial pH of 7.0 ± 0.1 to a final range of 5.5–6.0, indicating microbial activity associated with biodegradation. Statistical analysis (one-way ANOVA followed by Tukey’s HSD test, p < 0.05) showed that while pH decreased significantly over time for all strains (*p* < 0.05), differences in final pH values (day 35) among the strains were not significant (*p* > 0.05) (**Fig 4B**). In addition to growth and degradation efficiency, the microbial response to chemical species and bioavailable fractions in the medium likely influenced PET-MP biodegradation. Similar to observations reported by Pu et al. (2019), variations in bioavailable compounds can modulate microbial activities and community composition. The gradual pH decline and differences in degradation efficiency among strains suggest that metabolic activity and enzyme expression were affected by the chemical environment, including nutrient availability and PET-MP concentration. These results indicate that biodegradation is not solely determined by the presence of degradative enzymes, but also by microbial responses to chemical cues and stress conditions in the medium [50].

By day 7, the pH of PETKKU2 had decreased to 6.69 ± 0.02, eventually stabilizing at 6.50 ± 0.01 after 35 days. A similar trend was observed in PETKKU4, PETKKU6, PETKKU7, and PETKKU10, with final pH values of 6.48 ± 0.04, 6.31 ± 0.16, 6.47 ± 0.08, and 6.45 ± 0.01, respectively. The gradual pH declines from 7.0 to approximately 6.5 aligns with microbial biodegradation activity, likely due to the production of organic acids, a common characteristic of plastic biodegradation processes [49,51,52].

The degradation efficiency was evaluated by measuring the weight loss of PET-MP over 35 days (**Fig 4C**). The weight loss analysis showed varying degrees of degradation among the strains, with PETKKU2 exhibiting the highest degradation efficiency (6.07 ± 0.18%), followed by PETKKU10 (3.69 ± 0.86%), PETKKU6 (2.57 ± 0.69%), PETKKU7 (2.24 ± 0.91%), and PETKKU4 (2.15 ± 0.51%). Statistical analysis revealed that the degradation efficiency of PETKKU2 was significantly higher than all other strains (*p* < 0.01), while PETKKU10 demonstrated significantly higher degradation than PETKKU4, PETKKU6, and PETKKU7 (*p* < 0.05). No significant differences were observed among PETKKU4, PETKKU6, and PETKKU7 (*p* > 0.05). The relatively small SD for PETKKU2 indicates good reproducibility and consistency in its degradation performance.

Interestingly, PETKKU2 exhibited the highest PET-MP degradation (6.07 ± 0.18%) despite having the lowest OD₆₀₀ (0.160 ± 0.026), suggesting that efficient enzyme production and surface adhesion, rather than rapid biomass accumulation, play key roles in PET biodegradation. This inverse relationship between growth and degradation efficiency indicates a metabolic trade-off, where resources are preferentially allocated to enzyme secretion and substrate binding rather than cell division. Similar trends, where degradation activity does not correlate with cell density, have been reported in previous studies, [53,54], and may reflect differences in enzymatic efficiency, biofilm formation, and metabolic strategies among PET-degrading bacteria. Future proteomic or transcriptomic analyses could elucidate whether PETKKU2 exhibits higher expression of PET-degrading enzymes (e.g., esterases, lipases) compared to faster-growing strains. This weight reduction is attributed to microbial adhesion to the polymer surface and subsequent enzymatic degradation of PET-MP [51].

The statistical significance of the PET-MP degradation results was assessed using one-way analysis of variance (ANOVA) at a 95% confidence interval as described in the methods section. These statistical analyses support the selection of PETKKU2, PETKKU6, and PETKKU10 for further characterization and confirm that PETKKU2 demonstrates superior PET-MP degradation capabilities among the isolated strains. These findings align with previous studies on PET-MP degradation by various bacterial species [19,21,23].

This study underscores the complexity of microbial PET-MP degradation, showing that bacterial growth, pH fluctuations, and degradation efficiency do not always align. While PETKKU6 exhibited the most robust growth, it did not correlate with the highest degradation efficiency. This suggests that metabolic pathways and enzyme types are crucial for plastic breakdown. Weight loss analysis revealed PETKKU2, PETKKU6, and PETKKU10 as the most efficient degraders, with PETKKU2 being the most effective. These strains likely possess advanced enzymatic systems critical for PET-MP degradation.

Multiple lines of evidence confirmed the biodegradation potential of the selected strains. FTIR analysis revealed functional group modifications indicative of depolymerization and oxidation, including reductions in characteristic PET ester carbonyl (C = O, ~ 1715 cm ⁻ ¹) and aromatic C-O stretching (~1240 cm ⁻ ¹) peaks. SEM images showed pronounced surface roughening, erosion patterns, biofilm formation, and extensive microbial colonization of PET-MP surfaces. GC-MS analysis identified degradation-associated compounds including aldehydes, lactones, and hydrocarbons, indicating metabolic transformation of PET intermediates. These results collectively confirm the biodegradation potential of PETKKU2, PETKKU6, and PETKKU10, supporting their selection for further biochemical characterization and exploration of enzymatic pathways for plastic bioremediation.

### Identification and biochemical characterization of three isolated bacteria

Molecular identification of three bacterial isolates capable of degrading PET-MP was achieved via 16S rRNA gene sequencing. PCR amplification with universal primers (I-179R and I-179L) produced sequences of 1420 bp (PETKKU2), 1417 bp (PETKKU6), and 1383 bp (PETKKU10), which were submitted to GenBank with accession numbers PQ578631, PQ578632, and PQ578633, respectively. BLAST analysis identified all three isolates within the Firmicutes phylum, showing high sequence similarity to known species: PETKKU2 shared 99.71% similarity with *Paenibacillus naphthalenovorans* PR-N1, PETKKU6 shared 91.43% similarity with *Paenibacillus* sp. DRP1, and PETKKU10 shared 99.86% similarity with *Bacillus cereus* st2. Further phylogenetic analysis confirmed these relationships, with each isolate clustering with its respective closest match (S2–S4 Figs).

Phylogenetic analysis further confirmed these relationships, as PETKKU2 clustered with *P. naphthalenovorans* PR-N1 (S2 Fig), PETKKU6 with *Paenibacillus* sp. DRP1 (S3 Fig), and PETKKU10 with *B. cereus* st2 (S4 Fig). The Firmicutes group, known for its resilience to extreme environmental conditions [44], includes genera such as *Bacillus* and *Paenibacillus*, both capable of degrading plastics [55,56]. Notably, *P. naphthalenovorans* demonstrated superior PET-MP degradation efficiency compared to *B. cereus*. Similar to *Bacillus*, *Paenibacillus* can form biofilms [57] and produce surfactants [58], enhancing PET degradation in low-nutrient conditions. This study aligns with previous findings on the biodegradation capabilities of *Paenibacillus* strains, which are effective in remediating environments contaminated by petroleum and coal tar pollutants [58–60]. Despite limited research on *Paenibacillus* for PET-MP degradation, these findings position *Paenibacillus* as a promising candidate for bioremediation applications, paving the way for further exploration of its enzymatic pathways and advancing microbial plastic degradation technologies.

The biochemical characterization of *P. naphthalenovorans* PETKKU2, *Paenibacillus* sp. PETKKU6 and *B. cereus* PETKKU10 using HiBacillus™ kits (**Table 1**) highlight their potential for PET-MP degradation. All strains tested positive for citrate utilization, catalase activity, and arginine metabolism, indicating their ability to thrive in environments with limited carbon and nitrogen and their tolerance to oxidative stress [61,62]. These traits suggest improved degradation efficiency under harsh conditions, though differences in carbohydrate utilization point to varying metabolic flexibility, which could affect their degradation capabilities.

**Table 1:** Biochemical characterization of *P. naphthalenovorans* PETKKU2, *Paenibacillus* sp. PETKKU6 and *B. cereus* PETKKU10 using HiBacillus™.

Test	PETKKU2	PETKKU6	PETKKU10
**Malonate**	–	–	–
**Voges Proskauer’s**	–	–	–
**Citrate**	+	+	+
**ONPG**	–	+	+
**Nitrate**	+	–	–
**Catalase**	+	+	+
**Arginine**	+	+	+
**Sucrose**	+	–	–
**Mannitol**	+	–	–
**Glucose**	+	–	–
**Arabinose**	–	–	–
**Trehalose**	+	–	–

(+) Positive results and (-) Negative results.

PETKKU2 exhibited the broadest metabolic profile, utilizing sucrose, mannitol, glucose, and trehalose, and showed positive nitrate reduction. Nitrate reduction is crucial for anaerobic metabolism [63], enabling growth in oxygen-limited environments. This metabolic versatility enhances PETKKU2’s adaptability and contributes to its superior PET degradation ability. In contrast, PETKKU6 showed limited carbohydrate metabolism with positive o-Nitrophenyl-β-D-galactopyranoside (ONPG) activity but lacked nitrate reduction. PETKKU10 shared similarities with PETKKU6 but differed in nitrate reduction. Its limited sugar metabolism may reduce its degradation efficiency compared to PETKKU2. PETKKU2’s ability to utilize diverse carbohydrates and reduce nitrate provides a metabolic advantage, suggesting that it can produce a range of enzymes, enhancing its ability to degrade complex polymers like PET, even under low-oxygen conditions. These results align with the superior degradation performance discussed earlier.

While lipase production was used as an initial screening method to identify potential PET-MP degraders, our results revealed a complex relationship between lipase activity and actual degradation efficiency. Notably, PETKKU2 exhibited the highest PET-MP degradation (6.07 ± 0.18%) despite not showing the highest lipase activity (clear zone index of 1.43 ± 0.10 compared to 1.66 ± 0.06 for PETKKU4). This discrepancy suggests that although lipase production is an important factor in PET-MP degradation, it is not the sole determinant of degradation efficiency.

The superior performance of PETKKU2 may be attributed to its broader metabolic capabilities, as evidenced by its ability to utilize diverse carbohydrates (sucrose, mannitol, glucose, and trehalose) and reduce nitrate, traits not observed in other isolates (**Table 1**). Furthermore, genomic analysis of PETKKU2 revealed multiple biosynthetic gene clusters, including the NI-siderophore and ectoine biosynthesis clusters, which may contribute to its enhanced degradation capacity through improved stress adaptation and metal ion acquisition necessary for enzymatic function. These findings indicate that effective PET-MP biodegradation likely requires sophisticated enzymatic machinery beyond lipase activity alone, involving multiple metabolic pathways and adaptive mechanisms that allow the microorganism to effectively colonize and degrade the polymer under challenging conditions.

Comparing *P. naphthalenovorans* PR-N1 [64] from The Bacterial Diversity Metadatabase (BacDive) (https://bacdive.dsmz.de/strain/136597) with PETKKU2, both strains share several biochemical similarities, but differences in Arginine, Arabinose, and Trehalose metabolism highlight PETKKU2’s specific capabilities. PETKKU2’s ability to metabolize Trehalose but not Arabinose, while the BacDive strain can metabolize Arabinose, suggests differences in their metabolic flexibility. These variations could influence their suitability for different biodegradation processes, particularly for PET degradation under varying environmental conditions.

### Response surface optimization of PET-MP biodegradation

RSM optimization of PET-MP biodegradation by *P. naphthalenovorans* PETKKU2 yielded degradation efficiencies ranging from 2.85% to 11.15%. The second-order polynomial model demonstrated strong statistical significance (F = 21.14, p = 0.0003) with excellent predictive power (R² = 0.9645, adjusted R² = 0.9189). The model predicted maximum biodegradation at pH 7.5, 1.25 g/L NH₄NO₃, and 0.55% PET-MP, achieving 11.15% efficiency compared to 6.09% under non-optimized conditions (pH 7.0, 1.0 g/L NH₄NO₃, 1.0% PET-MP). Experimental validation near optimal conditions resulted in 9.48 ± 0.21% degradation, closely matching the predicted value of 10.38% (95% prediction interval: 8.42–12.34%).

**Fig 5:**
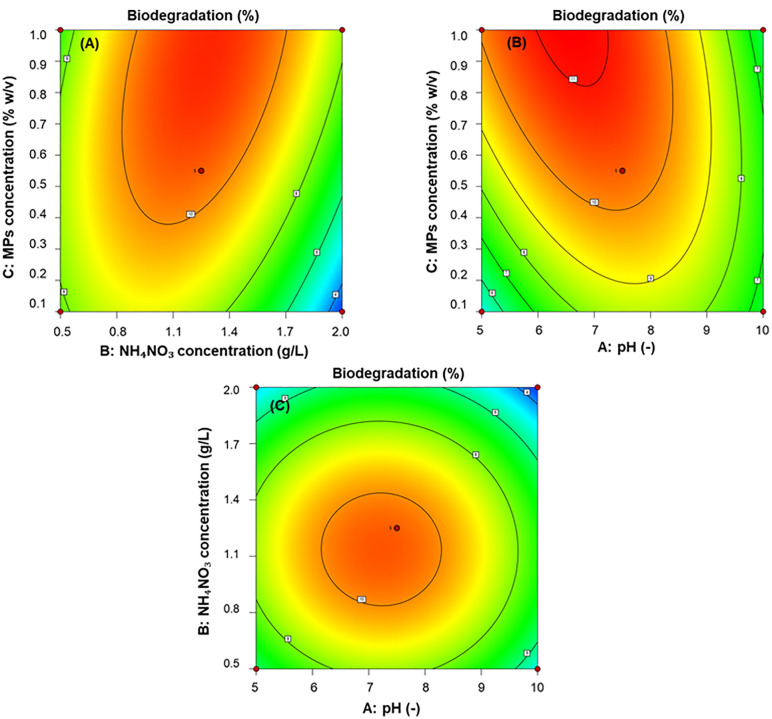
Response surface methodology optimization of Polyethylene terephthalate microplastics (PET-MP) biodegradation by *Paenibacillus naphthalenovorans* PETKKU2. Three-dimensional plots showing interactive effects of **(A)** NH₄NO₃ concentration (g/L) vs. PET-MP concentration (%), (B) pH vs. PET-MP concentration (%), and (C) pH vs. NH₄NO₃ concentration (g/L) on biodegradation efficiency after 35 days. Color gradient represents predicted degradation efficiency (%) from the Box-Behnken model (R² = 0.9645), with red indicating higher efficiency and blue indicating lower efficiency. Black dots (●) represent experimental data points near optimal conditions.

Among tested variables, PET-MP concentration had the greatest impact on biodegradation (p = 0.0033), followed by NH₄NO₃ concentration (p = 0.0116). Although pH alone was not statistically significant (p = 0.0635), its interactions with other factors showed important effects (S3 Table). **Significant** synergistic interactions were observed between PET-MP concentration and pH, as well as between PET-MP and NH₄NO₃ concentration (Fig 5). Residual analysis and diagnostic plots (Residuals vs. Predicted, Normal Plot of Residuals, predicted vs. actual; S5 Fig) were generated to confirm model adequacy, homoscedasticity, and normality of residuals. Quadratic terms for pH and NH₄NO₃ were also significant (p < 0.001), indicating non-linear effects on biodegradation. The regression equation was obtained from the RSM using a three-factor, three-level Box–Behnken design. The equation represents the relationship between biodegradation efficiency and the three independent variables: pH (A), NH₄NO₃ concentration (B, g/L), and PET-MP concentration (C, % w/v).

**Regression equation:**



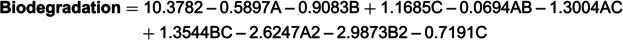



PET-MP concentration was the strongest factor influencing degradation, likely due to substrate-induced enzyme expression without inhibitory effects up to a certain threshold. In addition, variations in PET-MP concentration inherently influence the total available surface area for microbial and enzymatic interactions. Increased surface roughness and fragmentation observed in SEM images (**Fig 7**) likely enhanced enzyme accessibility, partially explaining the concentration-dependent biodegradation efficiency observed in this study [65,66]. NH₄NO₃ supported microbial metabolism and enhanced hydrolase activity, consistent with previous findings on nitrogen-mediated gene regulation in soil microbes [67,68]. Although pH did not show a significant linear effect, its interactions and quadratic terms highlight a narrow optimal pH range that supports enzyme stability and activity. In prior experiments, pH decreased from 7.0 to 6.5 during incubation, possibly due to organic acid production, a common byproduct of microbial plastic degradation [49]. Maintaining pH around 6.5–7.0 is important for enzymatic function, as deviations may reduce activity or cause repression.

The high R² value demonstrates the model’s robustness and suitability for guiding further optimization. These results emphasize the multifactorial nature of PET-MP biodegradation by PETKKU2. The data suggest involvement of inducible hydrolytic enzymes, such as polyethylene terephthalate hydrolase (PETase)/cutinase, whose expression depends on PET-MP concentration. Overall, RSM-based optimization confirms that PETKKU2 is a promising candidate for PET-MP bioremediation, especially under controlled conditions that enhance enzymatic expression and stability.

The optimized degradation efficiency (~11.15%) aligns with previous reports on PET-MP biodegradation resistance. For instance, *I. sakaiensis* degraded PET films by 75% in 70 days [18], while *Brucella intermedia* achieved 26% degradation in 30 days [69]. Our results are comparable to *B. cereus*, with 6.6% degradation [22], contributing valuable insight to the optimization-dependent nature of PET-MP biodegradation influenced by enzyme activity, substrate availability, and environmental factors. The mesophilic operating conditions (37 °C) and optimized degradation efficiency of 11.15% demonstrate practical potential for PET-MP bioremediation in temperate environments where energy-intensive thermophilic treatments would be less feasible.

### Characterization of PET-MP degradation by FTIR, SEM, and GC-MS

To confirm the PET-MP degradation efficiency discussed previously, FTIR and GC-MS analyses were performed on the three most efficient strains (PETKKU2, PETKKU6, and PETKKU10) to examine chemical modifications and degradation by-products. In addition, SEM analysis was conducted on the top-performing strain, PETKKU2, to observe surface morphological changes. Furthermore, the optimized biodegradation performance of PETKKU2 following RSM optimization was evaluated by FTIR and GC-MS. This comprehensive approach provides valuable insights into the physical and chemical transformations involved in PET-MP degradation and the mechanisms employed by these strains.

#### Structural and morphological changes in degraded PET-MP.

**Fig 6:**
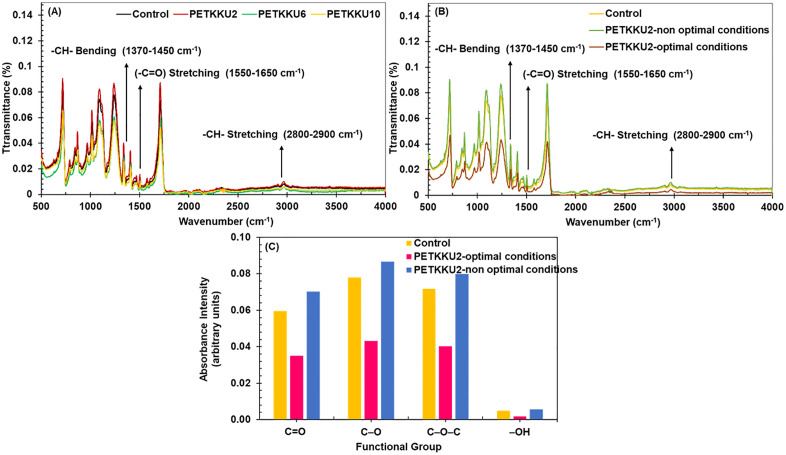
FTIR spectra of polyethylene terephthalate microplastics (PET-MP) after 35 days of treatment with *Paenibacillus naphthalenovorans* PETKKU2, *Paenibacillus* sp. PETKKU6, and *Bacillus cereus* PETKKU10, compared to untreated control. Changes in peak intensities and shifts corresponding to ester (C = O), hydroxyl **(O–H)**, and aliphatic (C–H) groups indicate microbial degradation **(A)**. FTIR spectra of PET-MP treated with PETKKU2 under optimized and non-optimized conditions compared to the control. Enhanced peak modifications under optimized conditions suggest increased oxidation and polymer chain scission **(B)**. Quantitative analysis of functional group changes under optimized and non-optimized conditions compared to the control **(C)**.

The FTIR spectrum of PET-MP treated with *P. naphthalenovorans* PETKKU2, *Paenibacillus* sp. PETKKU6 and *B. cereus* PETKKU10 showed significant changes compared to the untreated control, indicating microbial activity in degradation (**Fig 6A**). Hydroxyl (-OH) stretching at 3500–3300 cm ⁻ ¹ suggests the formation of alcohols or phenols, while carbonyl (-C = O) stretching at 1550–1650 cm ⁻ ¹ indicates aldehydes, ketones, or carboxylic acids. The decrease in PET-MP peaks, such as -CH- stretching at 2800–2900 cm ⁻ ¹ and -CH- bending at 1370–1450 cm ⁻ ¹, points to depolymerization and oxidative reactions, supporting microbial involvement in PET-MP breakdown [69,70]. These findings align with previous studies on polymer biodegradation [71–74], confirming the effectiveness of these strains in initiating PET-MP degradation.

**Fig 7:**
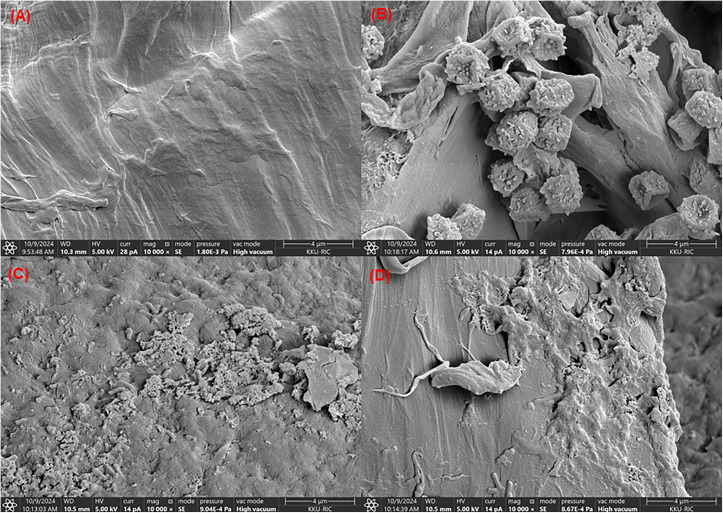
SEM micrographs of polyethylene terephthalate microplastics (PET-MP) (10,000 × magnification, scale bar = 4 µm): (A) Untreated control; (B-C) After 35 days of treatment with *Paenibacillus naphthalenovorans* PETKKU2 following gentle washing, showing biofilm formation and microbial colonization; (D) After stringent washing, revealing advanced surface erosion and polymer matrix degradation.

SEM images in **Fig 7** show significant degradation of PET-MP after 35 days of treatment with *P. naphthalenovorans* PETKKU2. The untreated PET-MP (**Fig 7A**) remains smooth, indicating no microbial activity, while treated samples (Fig 7B**–7D**) show surface alterations. **Fig 7B** displays roughened surfaces with microbial attachment and biofilm formation, while **Fig 7C** shows erosion and fragmentation, indicating enzymatic breakdown [66]. Fig 7D, after stringent washing to remove biofilm and cellular material, reveals advanced surface erosion with only minor residual fragments, confirming that the observed degradation represents actual polymer matrix breakdown rather than mere biofilm accumulation.

These findings align with previous studies on PET biodegradation [69,75], and confirm that PETKKU2 initiates degradation through biofilm formation and enzyme production, offering a promising solution for plastic waste management. Furthermore, studies indicate that *Paenibacillus* spp. can degrade low density polyethylene (LDPE) [76] and bioremediate pollution [58–60] via extracellular enzymes [77]. These findings indicate that *P. naphthalenovorans* PETKKU2 forms biofilms on the PET-MP surface, coinciding with surface erosion and fragmentation. While this does not definitively demonstrate that PET degradation is initiated by biofilm formation, it suggests that biofilm development likely facilitates enzyme-mediated polymer breakdown by promoting microbial attachment and enhancing local enzyme concentration. The observed PET-MP degradation likely involves complementary mechanisms. While SEM analysis revealed microbial attachment and biofilm formation (**Fig 7**), extracellular enzymes secreted by planktonic cells can also hydrolyze PET independently of direct cellular adhesion. Secreted depolymerases, hydrolases, and lipases [41,78,79], as well as enzyme adsorption onto polymer surfaces [80], can contribute to polymer chain scission and weight reduction without requiring intact cell-polymer contact. Consequently, PETKKU2 effectively degrades PET-MP through a combination of surface colonization and extracellular enzymatic activity, highlighting its potential for plastic waste management.

#### Metabolic intermediates and degradation pathway analysis.

GC–MS analysis identified several intermediate compounds in the culture supernatant collected after 35 days of PET-MP degradation, including lactones, aldehydes, alcohols, ketones, trienes, hydrocarbons, glycosides, and thiadiazoles (**Table 2**). It should be noted that metabolite analysis was performed only at the endpoint (day 35); no time-course sampling was conducted. Therefore, the proposed degradation pathway presented in Fig 9 is inferred based on these detected metabolites, genomic predictions of enzymatic capabilities, known PET depolymerization mechanisms, and literature reports, rather than on sequential tracking of intermediates over time. The pathway thus remains hypothetical and requires validation through time-resolved metabolite profiling and direct enzymatic assays*.*

**Table 2:** Metabolic profiles of PET-MP degradation products detected by GC–MS after 35 days of incubation with *P. naphthalenovorans* PETKKU2 (non-optimized), *Paenibacillus* sp. PETKKU6, and *B. cereus* PETKKU10. The table also includes data from PETKKU2 grown under optimized conditions.

Chemical Group	Compound Name	^a^PET KKU2	^b^PET KKU2	PET KKU6	PET KKU10
**Lactones**	α-Methyl-ε-caprolactone	D	D	ND	ND
	ε-Methyl-ε-caprolactone	D	D	ND	ND
	2-Butanoyloxytetrahydropyran	D	D	ND	ND
**Aldehydes**	2-Pentenal, (Z)-	D	D	ND	ND
**Trienes and Hydrocarbons**	[(4E)-1-tert-butylhexa-1,2,4-trienyl]benzene,	D	D	ND	ND
	15-methyltricyclo[6.5.2(13,14).0(7,15)] pentadeca-1,3,5,7,9,11,13-heptene	D	D	ND	ND
**Oxabicyclo**	13-Methyl-2-oxabicyclo[9.3.0]tetradec-1(11)-ene	D	D	ND	ND
**Tetracyclic Compounds**	7-n-Butyl-1-methyl-2,4,6,14-tetraoxapentacyclo [6.5.1.0(3,12).0(5,9).0(8,13)]tetradecane	D	D	ND	ND
**Glycosides**	Phenyl 4-[bis(ethoxycarbonyl) but-3-ynyl]-2,3,4-trideoxy-α,L-glcero-pent-2-enopyranoside	D	D	D	D
**Alcohols**	Butad-2,3-dien-1-ol,	ND	ND	D	ND
	1-(1-naphthalenyloxy)-3-(phenylseleno)-2-propanol	ND	ND	ND	D
**Ketones**	1-(4-Amino-3-butoxyphenyl)ethenone	ND	ND	D	ND
**Thiadiazoles**	5-(Phenoxy)methyl-2-amino-1,3,4-thiadiazoles	ND	ND	D	ND
**Heterocyclic Amine Oxide**	delta.(1)-4,5-Dihydropyrroline-1-oxide	D	ND	ND	ND
**Polyunsaturated Alcohol/Allene**	5-hydroxy-5-dideutero-1,2-pentadiene	D	ND	ND	ND
**Heterocyclic Compound (Triazole)**	1-Methyl-3-D-1,2,4-triazole	D	ND	ND	ND
**Aromatic Heterocycle**	6-(1’-t-Butyl-3’-phenylureido)-2,3,4,4a-tetrahydro-1H-pyrido[1,2-a]quinazoline-5-oxide	D	ND	ND	ND

D: Detected, ND: Not Detected; ^a^Optimized conditions, ^b^Non-optimized conditions.

*P. naphthalenovorans* PETKKU2 produced α-Methyl-ε-caprolactone, ε-Methyl-ε-caprolactone, 2-Butanoyloxytetrahydropyran, and 2-Pentenal (Z)-, while *Paenibacillus* sp. PETKKU6 yielded Butad-2,3-dien-1-ol and 1-(4-Amino-3-butoxyphenyl) ethanone. *B. cereus* PETKKU10 exclusively produced 1-(1-naphthalenyloxy)-3-(phenylseleno)- 2-propanol. The detection of these compounds provides insights into the stages of polymer breakdown and the biochemical mechanisms involved. These findings align with previous studies [81–83], highlighting that distinct bacterial strains produce unique metabolites at different stages of PET-MP degradation [84]. The results demonstrate the complexity of PET-MP degradation, with each strain contributing to specific stages by producing distinct intermediates. The identification of these metabolites provides valuable insights into the microbial pathways and mechanisms involved in PET-MP degradation.

#### Absence of canonical intermediates and proposed alternative pathway.

Previous studies have proposed the classical PET degradation pathway involving sequential hydrolysis of PET into BHET, MHET, and ultimately terephthalic acid (TPA) [14,85–87]. However, in the present study, GC–MS analysis of *P. naphthalenovorans* PETKKU2 revealed no detection of these canonical intermediates. Instead, cyclic lactone derivatives such as α-methyl-ε-caprolactone and ε-methyl-ε-caprolactone were identified, suggesting that PETKKU2 follows an alternative oxidative–hydrolytic degradation mechanism rather than the classical hydrolysis pathway. This proposed mechanism, illustrated in Fig 9, is further supported by genomic evidence indicating the presence of iron-dependent dioxygenases, monoacylglycerol lipases, and diverse carboxylesterases, but the absence of PETase/MHETase homologs.

We propose that PETKKU2 employs a combined oxidative attack and ester hydrolysis to generate oxidized short-chain fragments that undergo cyclization, bypassing BHET/MHET accumulation. The degradation process likely begins with bacterial adhesion to the PET-MP surface, facilitated by extracellular polymeric substances (EPS) [77], followed by coordinated enzymatic activities resembling the ethylene glycol (EG) degradation pathway of *Acetobacterium woodii* [88]. The resulting intermediates may subsequently enter the TCA cycle, contributing to energy generation and biosynthesis [77,84]. This multi-pathway mechanism could provide environmental benefits and enable production of valuable intermediates. Nevertheless, further experimental validation through enzyme characterization, time-course metabolite profiling, and targeted metabolite quantification is required to confirm this proposed mechanism.

#### Enhanced PET-MP biodegradation under optimized conditions.

**Improved structural degradation analysis by FTIR:** Under RSM-optimized conditions, FTIR analysis revealed enhanced structural degradation of PET-MP by *P. naphthalenovorans* PETKKU2. Compared to the untreated control and non-optimized conditions, significant reductions were observed in the ester carbonyl (~1714 cm ⁻ ¹) and aromatic C–H bending (~1408 cm ⁻ ¹) bands, indicating effective cleavage of ester linkages and partial disruption of the aromatic polymer backbone. Additionally, the broad O–H stretching band (~3450 cm ⁻ ¹) decreased, suggesting microbial assimilation of hydrolyzed products. New peaks observed at 3200–3600 cm ⁻ ¹ and near 1700 cm ⁻ ¹ corresponded to hydroxyl and carboxylic acid groups, implying oxidative modifications consistent with enzymatic activity (Fig 6B). These spectral shifts confirm intensified biodegradation of PET-MP under optimized conditions.

**Expanded metabolic profile and alternative pathways:** To further explore PET-MP degradation by PETKKU2, GC-MS analysis under optimized conditions revealed a broader range and higher diversity of PET-MP degradation metabolites compared to non-optimized treatments. While typical compounds such as lactones and aldehydes were still present, novel and structurally complex metabolites, e.g., polyunsaturated alcohols, oxidized cyclic ethers, and fused heterocycles like 1-methyl-3-D-1,2,4-triazole and 5-hydroxy-5-dideutero-1,2-pentadiene, were exclusively detected after optimization (Table 2). Notably, classical intermediates such as MHET were not detected under the experimental conditions. These observations may suggest the presence of alternative catabolic routes in PETKKU2, although the specific enzymes and pathways involved have not been experimentally characterized.

**Mechanistic shifts in PET-MP biodegradation through environmental optimization:** To gain deeper insight into the influence of environmental conditions, FTIR and GC-MS results were compared for PET-MP biodegradation by *P. naphthalenovorans* PETKKU2 under both optimized and non-optimized conditions. Complementary FTIR spectra showed a notable decrease in ester carbonyl (C = O) absorption (~1700 cm ⁻ ¹) by 41.2% under optimized conditions, and an increase of 17.9% under non-optimized conditions, relative to the control. Similar reductions were observed for C–O (44.6%) and C–O–C (44.0%) bonds under optimized conditions. Conversely, the –OH band (~3300 cm ⁻ ¹) intensity increased by 13.9% under non-optimized conditions but decreased by 62.6% under optimized conditions (Fig 6C).

**Qualitative structural changes observed by FTIR:** Under non-optimized conditions, relative intensities of ester carbonyl (C = O, ~ 1713 cm ⁻ ¹) and C–O–C (~1093 cm ⁻ ¹) bands slightly increased, which may reflect preferential degradation of amorphous regions, localized chain rearrangement, or surface compositional changes. Similar FTIR variations have been reported during polymer degradation [89]. However, FTIR provides only qualitative structural information; quantitative crystallinity determination would require DSC or XRD analysis. Under optimized conditions, the substantial 41% reduction in ester carbonyl intensity indicates extensive polymer backbone degradation. These spectral changes quantitatively confirm ester bond hydrolysis and oxidative modifications, complementing the GC-MS detection of degradation products and supporting the observed mechanistic shifts in PET-MP biodegradation by PETKKU2 [69]. The optimized treatment demonstrated a more extensive breakdown of ester bonds, greater oxidative transformation, and the formation of alternative degradation mechanism products do not present in the non-optimized setup. These included rare heterocycles and unsaturated intermediates, suggesting a shift in catabolic strategies (Fig 6B**–**6C).

Taken together with the expanded metabolic profile, these findings suggest that environmental optimization not only improves degradation efficiency but also alters the enzymatic and metabolic landscape. Genomic analysis further supported these observations, revealing the presence of genes encoding enzymes involved in depolymerization (lipases, carboxylesterases), aromatic ring oxidation (dioxygenases), and secondary metabolite biosynthesis. The absence of detectable MHET in optimized treatments may reflect rapid conversion or consumption of this intermediate rather than a definitive bypass of the classical PETase-MHETase pathway. While these observations suggest the possibility of alternative routes for PET degradation, the specific enzymatic mechanisms have not been experimentally confirmed. The resulting transformation of PET-MP involves hydrolysis, oxidation, ring cleavage, and assimilation of intermediates into central metabolic pathways such as the TCA cycle. This highlights the potential of PETKKU2 for bio-upcycling and plastic waste valorization under optimized conditions.

### Comparison of PET-MP degradation efficiencies with previous studies

Chemical recycling of PET typically requires harsh conditions (high temperatures, strong acids/bases, organic solvents) and is economically viable primarily for clean, bulk PET waste [90]. PET-MPs, however, are dispersed in environmental matrices, contaminated with various pollutants, and difficult to collect and process through conventional recycling infrastructure. Microbial degradation offers distinct advantages: it operates under mild conditions, requires no hazardous chemicals, can target contaminated or mixed plastic waste, and is potentially applicable in situ for bioremediation of microplastic pollution. Therefore, biological approaches complement chemical recycling by addressing scenarios where traditional methods are technically or economically impractical.

While most studies have focused on PET films and sheets, microplastics (PET-MP) remain underexplored despite their growing environmental impact. The physical form of PET significantly impacts biodegradation rates, with PET-MP representing a more challenging substrate due to higher crystallinity and a smaller surface area-to-volume ratio compared to films or sheets [84]. Additionally, experimental conditions vary widely across studies, including temperature, medium composition, and substrate preparation methods.

Previous studies demonstrate considerable variation in PET degradation efficiency. *I. sakaiensis* achieved the highest reported performance with 75% weight loss of PET films in 70 days [18], while *B. intermedia* IITR130 showed 26.06% degradation of sheets over 30 days [69]. Other notable performers include *Alcaligenes faecalis* (21% in 70 days) [70], *S. pyogenes* (3.846% in 30 days) [13], *M. arundinis* (3.0% in 14 days) [10], *Pseudomonas* sp. (3% in 56 days) [71], and *B. subtilis* B05 (0.3% in 15 days) [72]. For PET-MP specifically, *B. cereus* demonstrated 6.6% weight loss over 40 days [22], providing direct comparison for our study.

*P. naphthalenovorans* PETKKU2 achieved 6.07 ± 0.18% weight loss of PET-MP over 35 days under non-optimized conditions, closely matching *B. cereus* performance. Other isolated strains showed lower efficiencies: *Paenibacillus* sp. PETKKU6 (2.57 ± 0.69%) and *B. cereus* PETKKU10 (3.69 ± 0.86%). Under RSM-optimized conditions, PETKKU2 achieved 9.48% degradation efficiency, closely matching the model-predicted value of 10.38% (95% PI: 8.42–12.34%) and representing significant improvement toward the predicted maximum of 11.15% (**Table 3**).

**Table 3:** Comparison of PET-MP-degrading microorganisms for their efficiency in degrading PET.

Strains	PET	Time (day)	Weight Loss (%)	Ref.
*I. sakaiensis*	Film	70	75	[18]
*Brucella intermedia* IITR130	Sheet	30	26.06	[69]
*A. faecalis*	Sheet	70	21	[91]
*S. pyogenes*	Sheet	30	3.846	[20]
*M. arundinis*	Film	14	3.0	[17]
*Pseudomonas* sp.	Film	56	3	[92]
*B. cereus*	MP	40	6.6	[22]
*B. subtilis* B05	Sheet	15	0.3	[93]
***P. naphthalenovorans* PETKKU2**	**MP**	**35**	**9.48** ^*^	**This study (RSM-optimized)**
***P. naphthalenovorans* PETKKU2**	**MP**	**35**	**6.07**	**This study (non-optimized)**
*Paenibacillus* sp. PETKKU6			2.57	
*B. cereus* PETKKU10			3.69	

*Predicted maximum from RSM model = 11.15%; predicted value at validation point = 10.38% (95% PI: 8.42–12.34%).

Our stringent washing protocol (five washes with 2% SDS solution followed by multiple rinses) potentially yields more conservative measurements than some previous studies. Although degradation efficiency is lower than film-based studies, the results are significant for PET-MP degradation, a largely unexplored area. *P. naphthalenovorans* PETKKU2, isolated from plastic waste-contaminated soil, demonstrates real-world degradation potential. The strain’s mesophilic operation (37 °C) eliminates energy-intensive heating requirements of thermophilic degraders, making it suitable for integration into existing waste management infrastructure. The RSM optimization further validates the strain’s potential for scalable PET-MP bioremediation, establishing a foundation for sustainable plastic degradation processes with significant environmental benefits.

### Genomic analysis and degradation mechanisms

#### Genome characteristics and phylogenetic analysis.

**Fig 8:**
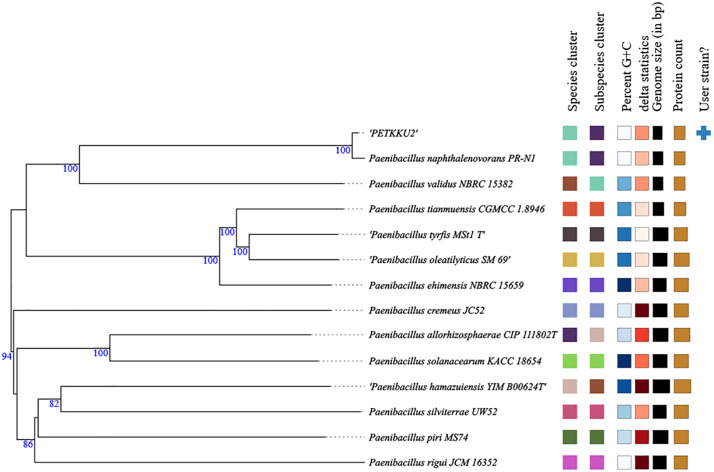
Phylogenetic tree of *Paenibacillus naphthalenovorans* PETKKU2 and related *Paenibacillus* type strains based on digital DNA-DNA hybridization (dDDH). The tree was inferred with FastME 2.1.6.1 from GBDP distances calculated from complete genome sequences, with branch lengths scaled in terms of GBDP distance formula d5. Numbers at nodes represent bootstrap support values (1000 replicates); only values >70% are shown. PETKKU2 shows 85.9% dDDH with *P. naphthalenovorans* PR-N1 (blue highlight), well above the 70% species delineation threshold [94], while all other *Paenibacillus* species show ≤15.5% dDDH (S5 Table). The scale bar represents GBDP distance units.

Whole-genome sequencing of *P. naphthalenovorans* PETKKU2 generated 6.802 million reads, resulting in a draft assembly of 5,074,235 bp (N50: 137,771 bp, GC: 50.04%, 97 contigs) (S4 Table). The genome has been deposited in NCBI GenBank under accession number JBLVQM000000000. Species-level identification of PETKKU2 was confirmed through phylogenetic analysis using digital DNA-DNA hybridization (dDDH) based on complete genome sequences. PETKKU2 exhibited 85.9% dDDH similarity with *P. naphthalenovorans* PR-N1, substantially exceeding the 70% threshold for species-level delineation [90] (Fig 8; S4 Table). In contrast, all other *Paenibacillus* type strains showed dDDH values ≤15.5%, clearly distinguishing PETKKU2 from other species within the genus. Maximum likelihood phylogenetic analysis further supported this classification with 100% bootstrap support. These genomic analyses confirmed PETKKU2 as *P. naphthalenovorans*. This relationship is relevant as PR-N1 degrades aromatic compounds in contaminated environments [64].

#### Genomic evidence for potential degradation enzymes.

Genome annotation revealed enzyme families potentially involved in PET degradation (S6 Table; Table 4). However, no enzymatic activities have been experimentally validated, and all assignments remain computational predictions.

**Table 4:** Predicted enzymes in the PET degradation pathway and correlations with experimental observations from PETKKU2.

Pathway Step	Predicted Enzyme Class	EC Number	Gene Count	Example Loci	Metal Cofactor	Experimental Evidence
**PET → BHET**	Monoacylglycerol lipases	3.1.1.23	2	KBGJGOPL_00244*, KBGJGOPL_03464	None	• Positive lipase screening • 41% reduction in C = O ester bonds at 1720 cm ⁻ ¹ • Progressive surface erosion • 11.09% weight loss
	Spore germination lipase	3.-.-.-	1	KBGJGOPL_02712	None	• Positive lipase screening • FTIR ester bond hydrolysis
	Putative esterases	3.1.-.-	3	KBGJGOPL_02931, KBGJGOPL_03559, KBGJGOPL_04417	None	• Correlation with weight loss
	Other ester hydrolases	3.1.1.*	13	Various (S6 Table)	Varies	• Supporting hydrolytic activity
**BHET → TPA + EG**	Same esterases	Various	~20 total	—	—	• BHET not detected (absence suggests rapid hydrolysis) • MHET not detected (bypasses classical pathway) • TPA inferred from aromatic metabolites
**TPA → Ring cleavage products**	Iron-dependent dioxygenases (metalloenzymes)	1.13.11.-	15	KBGJGOPL_03376, KBGJGOPL_02467, KBGJGOPL_01810, + 12 more	Fe² ⁺ /Fe³⁺	• Benzoic acid detected • Phthalic acid detected • Other aromatic intermediates • Consistent with PR-N1 aromatic degradation
**EG → Aldehydes**	Zinc-dependent alcohol dehydrogenases (metalloenzymes)	1.1.1.-	132	Various (S6 Table)	Zn² ⁺ , NAD⁺	• Multiple aldehyde products detected • Ethylene glycol not directly measured
**Aldehydes → Carboxylic acids**	Aldehyde dehydrogenases (metalloenzymes)	1.2.1.-	32	Various (S6 Table)	NAD ⁺ , Zn²⁺	• Various aldehydes detected • Carboxylic acid products detected
**Total**	**All enzyme classes**	—	**~199**	—	—	**Multiple correlative lines of evidence**

*Note: All enzyme assignments are computational predictions based on genome annotation (S6 Table). No enzymes have been heterologously expressed, purified, or biochemically characterized. Experimental evidence shows correlations but does not prove direct causation. Metal cofactors are predicted based on enzyme family characteristics and require experimental confirmation. Thermostable variant. BHET = bis(2-hydroxyethyl) terephthalate; TPA = terephthalic acid; EG = ethylene glycol

**Primary hydrolytic enzymes:** Genome annotation revealed two monoacylglycerol lipase genes (EC 3.1.1.23, loci KBGJGOPL_00244 and KBGJGOPL_03464), one spore germination lipase (EC 3.-.-.-; KBGJGOPL_02712), three putative esterases (EC 3.1.-.-), and thirteen additional carboxylic ester hydrolases (EC 3.1.1.-) that may hydrolyze ester bonds in the PET backbone (Table 4). Notably, KBGJGOPL_00244 encodes a thermostable monoacylglycerol lipase, potentially advantageous for applications at elevated temperatures. These findings correlate with positive lipase screening results (Fig 3), a 41.2% reduction in ester carbonyl groups at 1720 cm ⁻ ¹ under optimized conditions as observed by FTIR analysis (Fig 6C), and the 11.09% weight loss.

**Metalloenzymes predicted for secondary PET metabolite processing:** Genome analysis revealed iron-dependent dioxygenases (15 genes, EC 1.13.11.-) for aromatic ring cleavage, zinc-dependent alcohol dehydrogenases (132 genes, EC 1.1.1.-) for oxidizing alcohol intermediates, and aldehyde dehydrogenases (32 genes, EC 1.2.1.-, requiring NAD ⁺ /zinc) for aldehyde metabolism. These predictions are supported by: (i) related strain PR-N1’s naphthalene degradation capability [64]. and (ii) GC-MS detection of alcohol and aldehydes under both conditions (Tables 2 and 4). These metalloenzymes likely function in secondary metabolism of PET hydrolysis products rather than primary depolymerization. Experimental validation through proteomics and enzyme assays is needed to confirm their actual roles during PET-MP degradation.

Critically, no genes encoding specific PETase or MHETase enzymes characteristic of *I. sakaiensis* [14], triacylglycerol lipases (EC 3.1.1.3), classical carboxylesterases (EC 3.1.1.1), or cutinases (EC 3.1.1.74) were identified. PETKKU2 instead possesses monoacylglycerol lipases (EC 3.1.1.23) an enzyme class not previously characterized for PET degradation and diverse general esterases, representing a fundamentally distinct enzymatic repertoire. The well-characterized PET degrader *I. sakaiensis* employs specialized PETase (ISF6_4831) and MHETase (ISF6_0224) enzymes with high substrate specificity for PET [14], while PETKKU2 appears to utilize a broader enzymatic toolkit based on monoacylglycerol lipases. This difference in enzymatic strategy potentially explains the absence of MHET intermediates in our GC-MS analysis, as monoacylglycerol lipases may directly hydrolyze BHET to TPA and EG without the intermediate MHET step characteristic of the classical *I. sakaiensis* pathway.

Comparison with other *Paenibacillus* species reveals that while members of this genus possess diverse enzymatic capabilities including esterase activities [59], none have been characterized for PET degradation specifically. Recent advances in PET biodegradation include both protein-engineered variants [95,96] and naturally occurring thermostable enzymes like the PETase from *K. aridum* which operates at 70 °C [27]. In contrast, PETKKU2 represents a naturally evolved mesophilic system (37 °C) using monoacylglycerol lipases rather than specialized PETases or thermophilic enzymes, potentially offering a novel enzymatic approach to ambient temperature PET degradation.

#### Integration with experimental observations.

**Fig 9:**
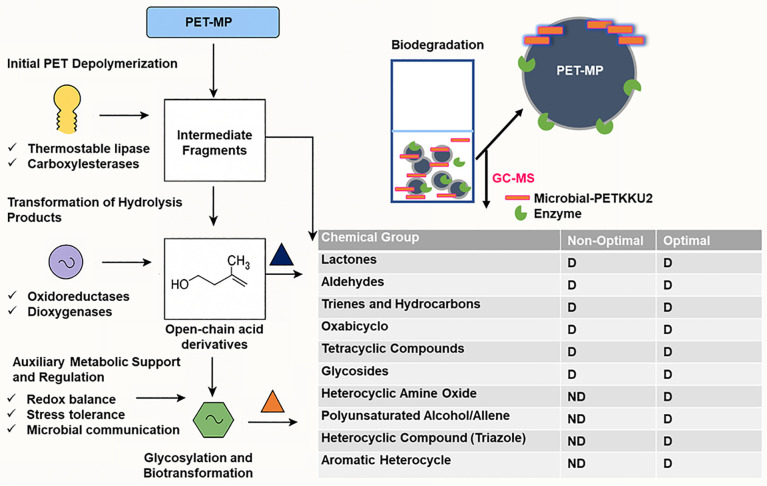
Proposed polyethylene terephthalate microplastics (PET-MP) degradation pathway by *Paenibacillus naphthalenovorans* PETKKU2. **(A)** Multi-step enzymatic mechanism showing depolymerization, oxidative ring cleavage, and biotransformation based on genomic analysis and GC-MS metabolite identification. Pathway steps are supported by detection of corresponding metabolites and genomic evidence of relevant enzymes. Orange and blue triangles indicate metabolites detected under optimized and non-optimized conditions, respectively.

The proposed degradation pathway (**Fig 9**) involves approximately 20 genes encoding ester hydrolases and 179 metalloenzymes potentially participating in PET breakdown (**Table 4**; S6 Table): 2 monoacylglycerol lipases, 1 spore germination lipase, ~ 17 other esterases, 15 iron-dependent dioxygenases, 132 zinc-dependent alcohol dehydrogenases, and 32 aldehyde dehydrogenases. These computational predictions show correlations with experimental observations as detailed in **Table 4**. The two monoacylglycerol lipases and other esterases align with positive lipase screening (**Fig 3**), the 41.2% reduction in ester carbonyl groups at 1720 cm ⁻ ¹ observed by FTIR analysis (**Fig 6C**), and the 11.09% weight loss achieved under optimized conditions. Iron-dependent dioxygenases correspond to the detection of aromatic degradation products, including benzoic acid and phthalic acid, in GC-MS analysis (**Table 2**). Zinc-dependent alcohol dehydrogenases and aldehyde dehydrogenases correlate with aldehyde intermediates detected by GC-MS. The SEM-observed progressive surface erosion (**Fig 7**) is consistent with predicted multi-enzyme hydrolytic activity.

However, as emphasized in **Table 4**, no enzymes have been expressed, purified, or experimentally validated for PET-degrading activity, and direct causation between predicted enzymes and observed degradation remains unproven. The complex metabolite profile (**Table 2**) correlates with the predicted enzymatic diversity shown in **Table 4**. The absence of both PETase/MHETase genes and MHET detection in GC-MS analysis suggests a possible alternative pathway: PET → Monoacylglycerol lipases/General esterases → BHET → Esterases → TPA + EG*,* bypassing MHET formation (**Fig 9**; **Table 4**). This alternative mechanism utilizes monoacylglycerol lipases rather than the specialized PETase/MHETase system or triacylglycerol lipases represents a fundamentally different approach from classical PET degradation pathways and expands our understanding of enzymatic strategies for synthetic polyester degradation.

### Limitations and future directions

All enzyme functions shown in **Table 4** and S5 Table are computationally predicted only; no direct enzymatic assays (e.g., PETase activity, ester hydrolysis with model substrates such as p-nitrophenyl esters, BHET hydrolysis assays) were performed. The proposed pathway illustrated in **Fig 9** and **Table 4** is hypothetical and unvalidated. PET crystallinity was not measured, which significantly affects degradation rates [97]. Metabolites were analyzed only at day 35, providing endpoint data rather than time-resolved monitoring of intermediate appearance and disappearance. This single-timepoint analysis prevents direct validation of the sequential degradation pathway and limits understanding of intermediate formation and consumption dynamics. Metalloenzyme cofactor requirements and expression levels during PET degradation remain unconfirmed.

Despite these limitations, this study provides the first genomic characterization of *P. naphthalenovorans* as a potential PET degrader, highlighting putative enzymatic systems and alternative degradation pathways. Future work should focus on: (1) heterologous expression and purification of candidate enzymes; (2) biochemical characterization including substrate specificity, kinetics, and direct activity assays; (3) time-course metabolite analysis (e.g., sampling at days 0, 7, 14, 21, 28, and 35) using GC-MS and/or LC-MS to track temporal dynamics of intermediate formation and consumption, validate the proposed sequential pathway, and identify rate-limiting steps; (4) quantitative PET crystallinity measurements (DSC/XRD) to assess preferential degradation of amorphous versus crystalline regions; (5) validation of metalloenzyme requirements; and (6) transcriptomic/proteomic analyses to identify enzymes upregulated during PET degradation.

## Conclusions

This study establishes *P. naphthalenovorans* PETKKU2 as a novel mesophilic PET-MP degrader, achieving 9.48% weight loss under optimized conditions (37 °C, 35 days), performance comparable to established degraders while eliminating energy-intensive heating requirements. Most significantly, comprehensive genomic and metabolomic analyses reveal an alternative degradation pathway utilizing monoacylglycerol lipases and general esterases rather than specialized PETase/MHETase enzymes, with MHET-independent hydrolysis representing a fundamentally distinct enzymatic strategy. These findings expand the known enzymatic repertoire for synthetic polyester degradation beyond the classical *I. sakaiensis* paradigm and demonstrate that effective PET biodegradation can occur through diverse biochemical routes. The mesophilic operation and moderate efficiency, while not yet competitive with engineered thermophilic systems, position PETKKU2 as a practical candidate for ambient-temperature bioremediation applications where energy input is limited. Critical next steps include heterologous expression and kinetic characterization of the predicted monoacylglycerol lipases (KBGJGOPL_00244, KBGJGOPL_03464), time-resolved metabolite profiling to validate the proposed MHET-independent pathway, and demonstration of degradation efficiency with environmentally weathered PET-MP rather than pristine pellets. Ultimately, understanding multiple biochemical strategies for PET degradation, both thermophilic/specialized and mesophilic/generalist, will inform development of flexible, context-appropriate solutions for the growing global challenge of microplastic pollution.

## Supporting information

S1 Fig FTIR spectra comparing polyethylene terephthalate microplastics before (PET-MP–not UV) and after UV sterilization (PET-MP–UV). The spectra exhibit the characteristic PET absorption peaks at around 1713 cm⁻¹ (C = O stretching), 1240 cm⁻¹ (C–O stretching), and 1090 cm⁻¹ (O–CH₂ stretching). No significant differences or new peaks were observed after UV exposure, indicating that the sterilization process did not noticeably alter the PET-MP chemical structure. Therefore, UV treatment was considered non-destructive for subsequent biodegradation experiments.
(DOCX)

S2 Fig Phylogenetic tree based on 16S rRNA gene sequences, showing the bacterial populations in Paenibacillus naphthalenovorans strain PETKKU2. The tree was constructed using 16S rRNA gene sequences retrieved from GenBank. Sequences were aligned with CLUSTALW in Unipro UGENE 51.0, and the phylogenetic tree was generated using Molecular Evolutionary Genetics Analysis Version 11 (MEGA 11). Bootstrap values based on 1000 replicates are shown above the branches.
(DOCX)

S3 Fig Phylogenetic tree based on 16S rRNA gene sequences, showing the bacterial populations in *Paenibacillus* sp. PETKKU6. The tree was constructed from 16S rRNA gene sequences retrieved from GenBank. Sequences were aligned using CLUSTALW in Unipro UGENE 51.0, and the phylogenetic tree was generated with Molecular Evolutionary Genetics Analysis Version 11 (MEGA 11). Bootstrap values based on 1000 replicates are shown above the branches.
(DOCX)

S4 Fig Phylogenetic tree based on 16S rRNA gene sequences, showing the bacterial populations in *Bacillus cereus* PETKKU10. The tree was constructed from 16S rRNA gene sequences retrieved from GenBank. Sequences were aligned using CLUSTALW in Unipro UGENE 51.0, and the phylogenetic tree was generated with Molecular Evolutionary Genetics Analysis Version 11 (MEGA 11). Bootstrap values based on 1000 replicates are shown above the branches.
(DOCX)

S5 Fig Diagnostic plots for response surface methodology (RSM) model validation: (A) Residuals vs. Predicted: Plot showing the distribution of residuals against predicted values. Residuals are randomly scattered around zero, indicating no obvious patterns and suggesting homoscedasticity. (B) Normal Plot of Residuals: Normal probability plot of residuals demonstrating approximate normality, supporting the assumption of normally distributed errors in the model. (D) Predicted vs. Actual: Comparison of predicted versus actual response values. Data points closely align along the 45° line, indicating good agreement between the model predictions and experimental observations.
(DOCX)

S1 Table The physical characteristics of soil samples collected from an open dump landfill, Khon Kaen province, Thailand. (DOCX)

S2 Table Morphology of polyethylene terephthalate microplastic (PET-MP) degrading microorganisms isolated from microbial consortium obtained from an open dump landfill. (DOCX)

S3 Table Analysis of variance (ANOVA) for the quadratic model used in the biodegradation of PET-MPs by *Paenibacillus naphthalenovorans* strain PETKKU2. (DOCX)

S4 Table Genome assembly statistics and sequencing quality metrics of *Paenibacillus naphthalenovorans* PETKKU2. The table presents sequencing read quality, assembly statistics, and genome characteristics including contig sizes, GC content, and assembly quality indicators. Total reads and bases are shown in millions (M) and gigabases (G), respectively. Contig lengths and N statistics are presented in base pairs (bp).
(DOCX)

S5 Table Genomic characteristics of type strains within the *Paenibacillus* genus. The table presents species cluster information, strain designations, repository information, authority references, synonymous taxon names, genome size (base pairs), GC content (%), and number of protein-coding genes for 13 different *Paenibacillus* type strains.
(DOCX)

S6 Table Genes are associated with PET degradation in the PETKKU2 genome. (DOCX)
