# Supplementary material for: Biodegradation of polyethylene terephthalate microplastics by Paenibacillus naphthalenovorans PETKKU2: Response surface optimization and genomic evidence for an alternative degradation mechanism
Source: PLoS One. 2026 Feb 4;21(2):e0341623. doi: 10.1371/journal.pone.0341623 (PMC12871986; doi:10.1371/journal.pone.0341623)
Supplement: S1 Fig — The spectra exhibit the characteristic PET absorption peaks at around 1713 cm⁻¹ (C = O stretching), 1240 cm⁻¹ (C–O stretching), and 1090 cm⁻¹ (O–CH₂ stretching). No significant differences or new peaks were observed after UV exposure, indicating that the sterilization process did not noticeably alter the PET-MP chemical structure. Therefore, UV treatment was considered non-destructive for subsequent biodegradation experiments. (DOCX) [file pone.0341623.s001.docx]

**Supplementary Fig. S1**


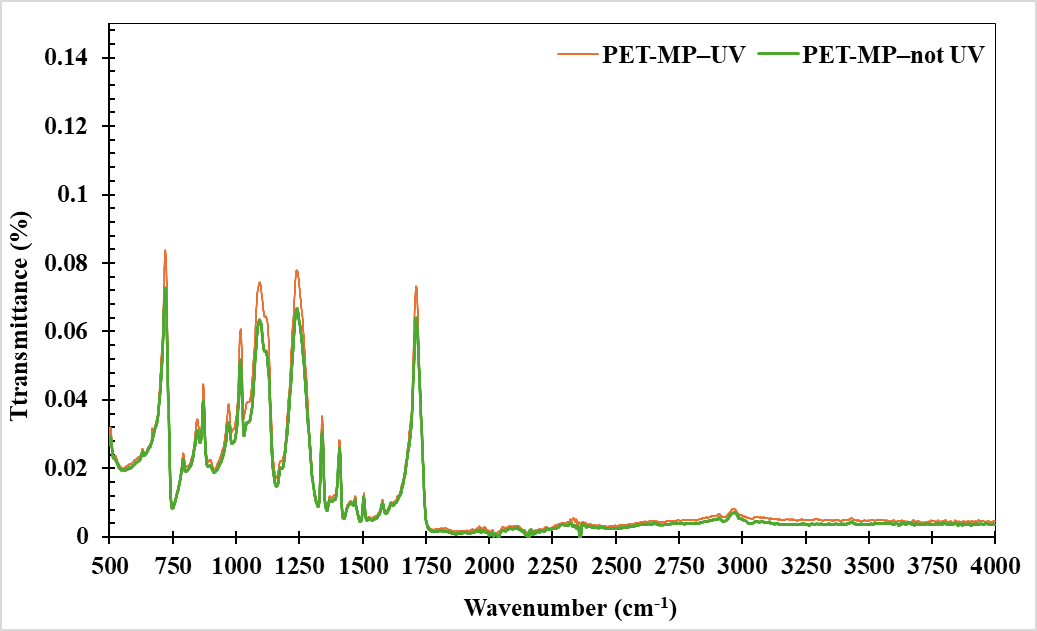


**Fig. S1** FTIR spectra comparing polyethylene terephthalate microplastics before (PET-MP–not UV) and after UV sterilization (PET-MP–UV). The spectra exhibit the characteristic PET absorption peaks at around 1713 cm⁻¹ (C=O stretching), 1240 cm⁻¹ (C–O stretching), and 1090 cm⁻¹ (O–CH₂ stretching). No significant differences or new peaks were observed after UV exposure, indicating that the sterilization process did not noticeably alter the PET-MP chemical structure. Therefore, UV treatment was considered non-destructive for subsequent biodegradation experiments.
