# Supplementary material for: Biodegradation of polyethylene terephthalate microplastics by Paenibacillus naphthalenovorans PETKKU2: Response surface optimization and genomic evidence for an alternative degradation mechanism
Source: PLoS One. 2026 Feb 4;21(2):e0341623. doi: 10.1371/journal.pone.0341623 (PMC12871986; doi:10.1371/journal.pone.0341623)
Supplement: S2 Fig — The tree was constructed using 16S rRNA gene sequences retrieved from GenBank. Sequences were aligned with CLUSTALW in Unipro UGENE 51.0, and the phylogenetic tree was generated using Molecular Evolutionary Genetics Analysis Version 11 (MEGA 11). Bootstrap values based on 1000 replicates are shown above the branches. (DOCX) [file pone.0341623.s002.docx]

**Supplementary Fig. S2**


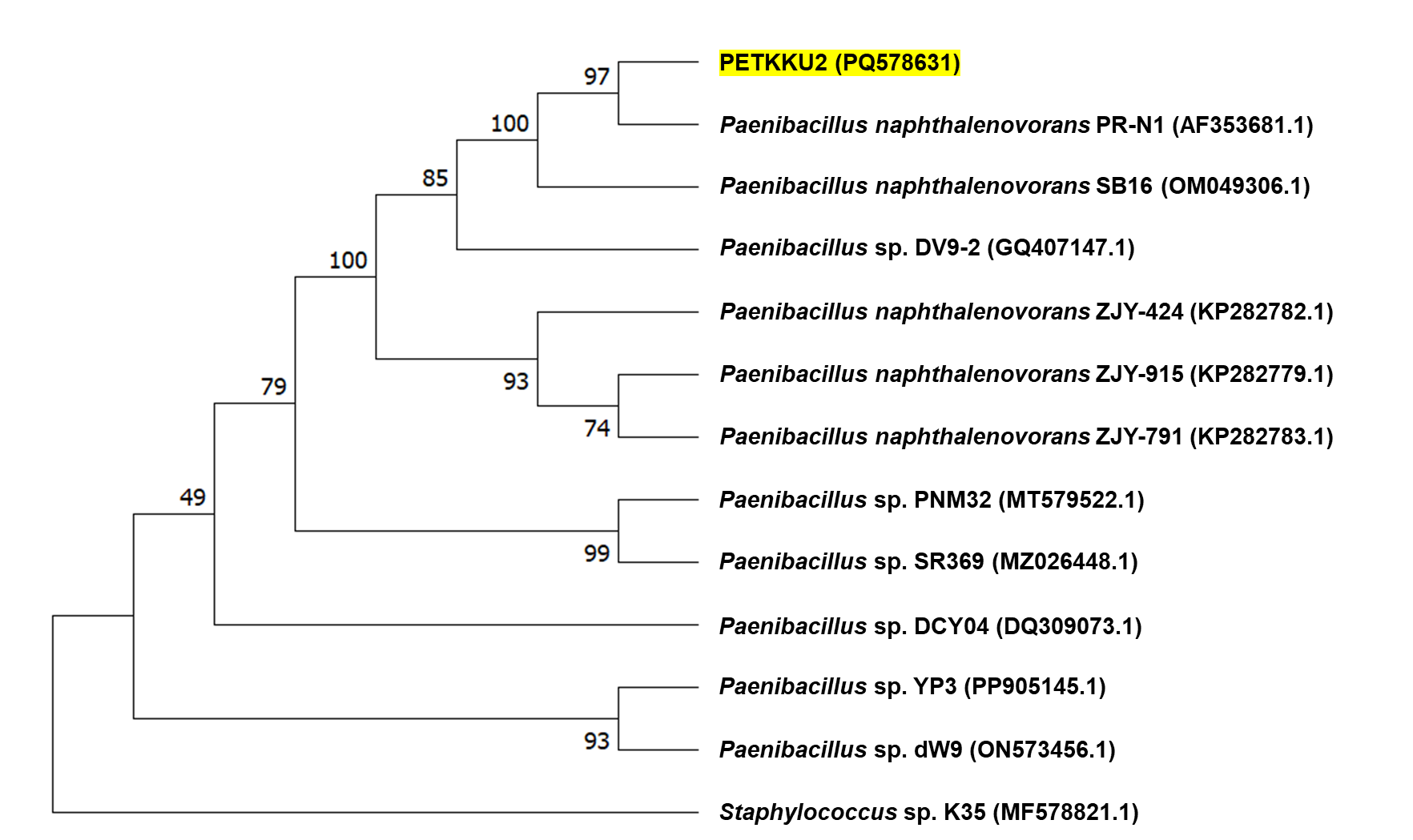


**Fig. S2** Phylogenetic tree based on 16S rRNA gene sequences, showing the bacterial populations in *Paenibacillus naphthalenovorans* strain PETKKU2. The tree was constructed using 16S rRNA gene sequences retrieved from GenBank. Sequences were aligned with CLUSTALW in Unipro UGENE 51.0, and the phylogenetic tree was generated using Molecular Evolutionary Genetics Analysis Version 11 (MEGA 11). Bootstrap values based on 1000 replicates are shown above the branches.
