# Supplementary material for: Biodegradation of polyethylene terephthalate microplastics by Paenibacillus naphthalenovorans PETKKU2: Response surface optimization and genomic evidence for an alternative degradation mechanism
Source: PLoS One. 2026 Feb 4;21(2):e0341623. doi: 10.1371/journal.pone.0341623 (PMC12871986; doi:10.1371/journal.pone.0341623)
Supplement: S5 Fig — Residuals are randomly scattered around zero, indicating no obvious patterns and suggesting homoscedasticity. (B) Normal Plot of Residuals: Normal probability plot of residuals demonstrating approximate normality, supporting the assumption of normally distributed errors in the model. (D) Predicted vs. Actual: Comparison of predicted versus actual response values. Data points closely align along the 45° line, indicating good agreement between the model predictions and experimental observations. (DOCX) [file pone.0341623.s005.docx]

**Supplementary Fig. S5**

**
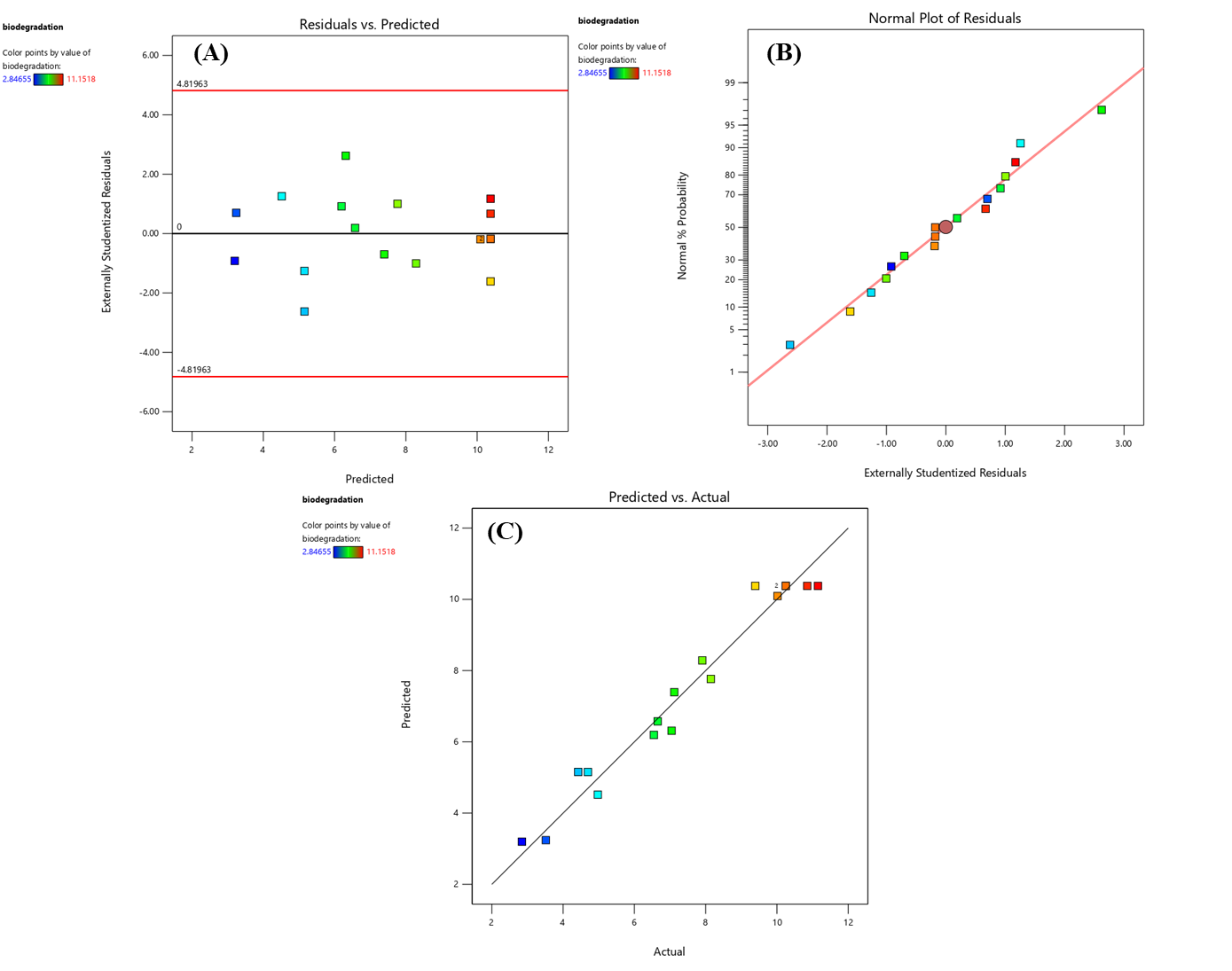
**

**Fig. 5** Diagnostic plots for response surface methodology (RSM) model validation: (A) Residuals vs. Predicted: Plot showing the distribution of residuals against predicted values. Residuals are randomly scattered around zero, indicating no obvious patterns and suggesting homoscedasticity. (B) Normal Plot of Residuals: Normal probability plot of residuals demonstrating approximate normality, supporting the assumption of normally distributed errors in the model. (D) Predicted vs. Actual: Comparison of predicted versus actual response values. Data points closely align along the 45° line, indicating good agreement between the model predictions and experimental observations.
