# Supplementary material for: Biodegradation of polyethylene terephthalate microplastics by Paenibacillus naphthalenovorans PETKKU2: Response surface optimization and genomic evidence for an alternative degradation mechanism
Source: PLoS One. 2026 Feb 4;21(2):e0341623. doi: 10.1371/journal.pone.0341623 (PMC12871986; doi:10.1371/journal.pone.0341623)
Supplement: S1 Table — (DOCX) [file pone.0341623.s006.docx]

**Supplementary Table S1**

**Table S1** The physical characteristics of soil samples collected from an open dump landfill, Khon Kaen province, Thailand

| **Point** | **Coordinate** | | **pH** | **Temperature (°C)** | **Physical characteristic** |
| --- | --- | --- | --- | --- | --- |
|  | Latitude | Longitude |  |  |  |
| 1 | 265843 | 1835897 | 6 | 29 | Black, Loam soil |
| 2 | 265971 | 1835913 | 5 | 32 | Black, Loam soil |
| 3 | 266041 | 1835945 | 5 | 30 | Black, Loam soil |
| 4 | 266305 | 1835970 | 6 | 32 | Black, Loam soil |
| 5 | 266305 | 1835970 | 6 | 31 | Black, Loam soil |
| 6 | 266305 | 1835970 | 10 | 35 | Black, Loam soil |
