# Supplementary material for: Biodegradation of polyethylene terephthalate microplastics by Paenibacillus naphthalenovorans PETKKU2: Response surface optimization and genomic evidence for an alternative degradation mechanism
Source: PLoS One. 2026 Feb 4;21(2):e0341623. doi: 10.1371/journal.pone.0341623 (PMC12871986; doi:10.1371/journal.pone.0341623)
Supplement: S2 Table — (DOCX) [file pone.0341623.s007.docx]

**Supplementary Table S2**

**Table S2** Morphology of polyethylene terephthalate microplastic (PET-MP) degrading microorganisms isolated from microbial consortium obtained from an open dump landfill.

| **Designated as** | **Morphological characteristics** | |
| --- | --- | --- |
|  | **Colony morphology** | **Cell morphology** |
| PETKKU1 | small to medium, white, lens-shaped | Gram-positive |
| PETKKU2 | small, pale yellow or cream-coloured, lens-shaped | Gram-positive |
| PETKKU3 | small, white, lens-shaped | Gram-positive |
| PETKKU4 | small, pale yellow or cream-coloured, lens-shaped | Gram-positive |
| PETKKU5 | small, cream, lens-shaped | Gram-positive |
| PETKKU6 | small to medium, white, lens-shaped to smooth | Gram-positive |
| PETKKU7 | small, white, lens-shaped | Gram-positive |
| PETKKU8 | small, yellow, lens-shaped to smooth | Gram-positive |
| PETKKU9 | small, white, smooth | Gram-positive |
| PETKKU10 | small to medium, white, lens-shaped to smooth | Gram-positive |
