# Supplementary material for: Biodegradation of polyethylene terephthalate microplastics by Paenibacillus naphthalenovorans PETKKU2: Response surface optimization and genomic evidence for an alternative degradation mechanism
Source: PLoS One. 2026 Feb 4;21(2):e0341623. doi: 10.1371/journal.pone.0341623 (PMC12871986; doi:10.1371/journal.pone.0341623)
Supplement: S3 Table — (DOCX) [file pone.0341623.s008.docx]

**Supplementary Table S3**

**Table S3** Analysis of variance (ANOVA) for the quadratic model used in the biodegradation of PET microplastics by *Paenibacillus naphthalenovorans* strain PETKKU2.

| **Source** | **Sum of Squares** | **Df** | **Mean Square** | **F-value** | **p-value** |  |
| --- | --- | --- | --- | --- | --- | --- |
| **Model** | 109.18 | 9 | 12.13 | 21.14 | 0.0003 | **Significant** |
| A | 2.78 | 1 | 2.78 | 4.85 | 0.0635 |  |
| B | 6.6 | 1 | 6.6 | 11.5 | 0.0116 |  |
| C | 10.92 | 1 | 10.92 | 19.04 | 0.0033 |  |
| AB | 0.0192 | 1 | 0.0192 | 0.0335 | 0.8599 |  |
| AC | 6.76 | 1 | 6.76 | 11.79 | 0.0109 |  |
| BC | 7.34 | 1 | 7.34 | 12.79 | 0.009 |  |
| A² | 29.01 | 1 | 29.01 | 50.56 | 0.0002 |  |
| B² | 37.58 | 1 | 37.58 | 65.49 | < 0.0001 |  |
| C² | 2.18 | 1 | 2.18 | 3.79 | 0.0924 |  |
| **Residual** | 4.02 | 7 | 0.5738 |  |  |  |
| Lack of Fit | 2.19 | 3 | 0.7304 | 1.6 | 0.3223 | **not significant** |
| Pure Error | 1.82 | 4 | 0.4562 |  |  |  |
| **Cor Total** | 113.2 | 16 |  |  |  |  |

**Note:** A = pH, B = ammonium nitrate concentration (g/L), C = PET-MP concentration (%w/v). Significant model terms are indicated by p < 0.05.
