# Supplementary material for: Biodegradation of polyethylene terephthalate microplastics by Paenibacillus naphthalenovorans PETKKU2: Response surface optimization and genomic evidence for an alternative degradation mechanism
Source: PLoS One. 2026 Feb 4;21(2):e0341623. doi: 10.1371/journal.pone.0341623 (PMC12871986; doi:10.1371/journal.pone.0341623)
Supplement: S4 Table — The table presents sequencing read quality, assembly statistics, and genome characteristics including contig sizes, GC content, and assembly quality indicators. Total reads and bases are shown in millions (M) and gigabases (G), respectively. Contig lengths and N statistics are presented in base pairs (bp). (DOCX) [file pone.0341623.s009.docx]

**Supplementary Table S4**

**Table S4** Genome assembly statistics and sequencing quality metrics of *Paenibacillus naphthalenovorans* PETKKU2. The table presents sequencing read quality, assembly statistics, and genome characteristics including contig sizes, GC content, and assembly quality indicators. Total reads and bases are shown in millions (M) and gigabases (G), respectively. Contig lengths and N statistics are presented in base pairs (bp).

| **Quality of the sequencing reads** | |
| --- | --- |
| Total reads (M) | 6.802 |
| Total bases (G) | 1.003 |
| CDS | 4917 |
| rRNA | 1 |
| tRNA | 77 |
| tmRNA | 1 |
| **Quality assessment of genome assemblies*** | |
| Contigs | 97 |
| Largest contig | 899235 |
| Total length (bp) | 5074235 |
| N50 | 137771 |
| N75 | 71578 |
| L50 | 10 |
| L75 | 23 |
| GC (%) | 50.04 |

*All statistics are based on contigs of size >= 500 bp, unless otherwise noted (e.g., "# contigs (>= 0 bp)" and "Total length (>= 0 bp)" include all contigs)
