# Supplementary material for: Biodegradation of polyethylene terephthalate microplastics by Paenibacillus naphthalenovorans PETKKU2: Response surface optimization and genomic evidence for an alternative degradation mechanism
Source: PLoS One. 2026 Feb 4;21(2):e0341623. doi: 10.1371/journal.pone.0341623 (PMC12871986; doi:10.1371/journal.pone.0341623)
Supplement: S5 Table — The table presents species cluster information, strain designations, repository information, authority references, synonymous taxon names, genome size (base pairs), GC content (%), and number of protein-coding genes for 13 different Paenibacillus type strains. (DOCX) [file pone.0341623.s010.docx]

**Supplementary Table S5**

**Table S5** Genomic characteristics of type strains within the *Paenibacillus* genus. The table presents species cluster information, strain designations, repository information, authority references, synonymous taxon names, genome size (base pairs), GC content (%), and number of protein-coding genes for 13 different *Paenibacillus* type strains.

| **Species** | **Accession No.** | **dDDH (%)** | **Size (bp)** | **G+C (%)** | **No. proteins** | **Reference** |
| --- | --- | --- | --- | --- | --- | --- |
| **PETKKU2** | **PQ578631** | **100.0** | **5,074,235** | **50.04** | **4971** | **This study** |
| *Paenibacillus naphthalenovorans* PR-N1 | PRJEB15954 | 85.9 | 5,289,735 | 50.05 | 5176 | (Daane et al., 2002) |
| *Paenibacillus validus* NBRC 15382 | PRJDB1368 | 15.5 | 5,530,436 | 52.19 | 5276 | (Nakamura, 1984) |
| *Paenibacillus tianmuensis* CGMCC 1.8946 | PRJEB15998 | 15.0 | 6,019,818 | 52.48 | 5568 | (Wu et al., 2011) |
| *Paenibacillus ehimensis* NBRC 15659 | PRJDB380 | 14.8 | 7,467,279 | 54.24 | 6811 | (Kuroshima et al., 1996) |
| *Paenibacillus oleatilyticus* SM 69 | PRJNA737459 | 14.8 | 7,884,376 | 53.16 | 7216 | (Chauhan et al., 2022) |
| *Paenibacillus tyrfis* MSt1 T | PRJNA246682 | 14.6 | 8,020,408 | 53.02 | 6455 | (Aw et al., 2016) |
| *Paenibacillus allorhizosphaerae* CIP 111802T | PRJEB45811 | 14.0 | 8,159,620 | 51.2 | 7367 | (Kampfer et al., 2021) |
| *Paenibacillus cremeus* JC52 | PRJNA556545 | 13.9 | 7,557,764 | 50.72 | 6701 | (Kim et al., 2022) |
| *Paenibacillus solanacearum* KACC 18654 | PRJEB45811 | 13.9 | 7,592,452 | 53.99 | 6667 | (Cho et al., 2017) |
| *Paenibacillus silviterrae* UW52 | PRJNA971768 | 13.6 | 7,436,003 | 51.65 | 6512 | (Lee et al., 2024) |
| *Paenibacillus rigui* JCM 16352 | PRJNA393491 | 13.2 | 7,172,832 | 50.27 | 6159 | (Baik et al., 2011) |
| *Paenibacillus piri* MS74 | PRJNA526414 | 13.2 | 7,965,182 | 51.04 | 6809 | (Trinh & Kim, 2020) |
| *Paenibacillus hamazuiensis* YIM B00624 | PRJNA224116 | 13.1 | 8,795,693 | 53.4 | 7632 | (Wang et al., 2022) |

**Reference**

Aw, Y. K., Ong, K. S., Lee, L. H., Cheow, Y. L., Yule, C. M., & Lee, S. M. (2016). Newly Isolated *Paenibacillus tyrfis* sp. nov., from Malaysian Tropical Peat Swamp Soil with Broad Spectrum Antimicrobial Activity. *Front Microbiol*, *7*, 219. https://doi.org/10.3389/fmicb.2016.00219

Baik, K. S., Lim, C. H., Choe, H. N., Kim, E. M., & Seong, C. N. (2011). *Paenibacillus rigui* sp. nov., isolated from a freshwater wetland. *Int J Syst Evol Microbiol*, *61*(3), 529–534. https://doi.org/10.1099/ijs.0.021485-0

Chauhan, N. S., Joseph, N., Shaligram, S., Chavan, N., Joshi, A., Dhotre, D., Lodha, T., & Shouche, Y. (2022). *Paenibacillus oleatilyticus* sp. nov., isolated from soil. *Arch Microbiol*, *204*(8), 516. https://doi.org/10.1007/s00203-022-03116-0

Cho, H., Heo, J., Ahn, J. H., Weon, H. Y., Kim, J. S., Kwon, S. W., & Kim, S. J. (2017). *Paenibacillus solanacearum* sp. nov., isolated from rhizosphere soil of a tomato plant. *Int J Syst Evol Microbiol*, *67*(12), 5046–5050. https://doi.org/10.1099/ijsem.0.002410

Daane, L. L., Harjono, I., Barns, S. M., Launen, L. A., Palleron, N. J., & Haggblom, M. M. (2002). PAH-degradation by *Paenibacillus* spp. and description of *Paenibacillus naphthalenovorans* sp. nov., a naphthalene-degrading bacterium from the rhizosphere of salt marsh plants. *Int J Syst Evol Microbiol*, *52*(1), 131–139. https://doi.org/10.1099/00207713-52-1-131

Kampfer, P., Busse, H. J., McInroy, J. A., Clermont, D., Criscuolo, A., & Glaeser, S. P. (2021). *Paenibacillus allorhizosphaerae* sp. nov., from soil of the rhizosphere of Zea mays. *Int J Syst Evol Microbiol*, *71*(10), 5051. https://doi.org/10.1099/ijsem.0.005051

Kim, J., Chhetri, G., Kim, I., So, Y., & Seo, T. (2022). *Paenibacillus agilis* sp. nov., *Paenibacillus cremeus* sp. nov. and *Paenibacillus terricola* sp. nov., isolated from rhizosphere soils. *Int J Syst Evol Microbiol*, *72*(12), 5640. https://doi.org/10.1099/ijsem.0.005640

Kuroshima, K. I., Sakane, T., Takata, R., & Yokota, A. (1996). *Bacillus ehimensis* sp. nov. and *Bacillus chitinolyticus* sp. nov., new chitinolytic members of the genus *Bacillus*. *Int. J. Syst. Bacteriol.*, *46*(1), 76–80. https://doi.org/10.1099/00207713-46-1-76

Lee, H., Chaudhary, D. K., Lee, K. E., Cha, I. T., Chi, W. J., & Kim, D. U. (2024). *Microbacterium humicola* sp. nov., *Microbacterium terrisoli* sp. nov., *Paenibacillus pedocola* sp. nov., *Paenibacillus silviterrae* sp. nov., *Flavobacterium terrisoli* sp. nov., and *Aquabacterium humicola* sp. nov., isolated from soil. *Int J Syst Evol Microbiol*, *74*(8), 6486. https://doi.org/10.1099/ijsem.0.006486

Nakamura, L. K. (1984). *Bacillus amylolyticus* sp. nov., nom. rev., *Bacillus lautus* sp. nov., nom. rev., *Bacillus pabuli* sp. nov., nom. rev., and *Bacillus validus* sp. nov., nom. rev. *Int. J. Syst. Bacteriol.*, *34*(2), 224–226. https://doi.org/10.1099/00207713-34-2-224

Trinh, N. H., & Kim, J. (2020). *Paenibacillus piri* sp. nov., isolated from urban soil. *Int J Syst Evol Microbiol*, *70*(1), 656–661. https://doi.org/10.1099/ijsem.0.003811

Wang, J., Deng, M., Zhou, E. M., Ran, L., Miao, C. P., Li, Y. Q., Ding, J. M., & Tang, S. K. (2022). *Paenibacillus hamazuiensis* sp. nov., a bacterium isolated from Hamazui hot spring in Yunnan province, south-west China. *Arch Microbiol*, *204*(11), 676. https://doi.org/10.1007/s00203-022-03282-1

Wu, X., Fang, H., Qian, C., Wen, Y., Shen, X., Li, O., & Gao, H. (2011). *Paenibacillus tianmuensis* sp. nov., isolated from soil. *Int J Syst Evol Microbiol*, *61*(5), 1133–1137. https://doi.org/10.1099/ijs.0.024109-0
