# Supplementary material for: Biodegradation of polyethylene terephthalate microplastics by Paenibacillus naphthalenovorans PETKKU2: Response surface optimization and genomic evidence for an alternative degradation mechanism
Source: PLoS One. 2026 Feb 4;21(2):e0341623. doi: 10.1371/journal.pone.0341623 (PMC12871986; doi:10.1371/journal.pone.0341623)
Supplement: S6 Table — (DOCX) [file pone.0341623.s011.docx]

**Supplementary Table S6**

**Table S6** Genes are associated with PET degradation in the PETKKU2 genome.

| **Category** | **Enzyme** | **Gene** | **EC Number** | **Role in Pathway** |
| --- | --- | --- | --- | --- |
| Primary PET Degradation | | | | |
|  | Lipase | - | EC 3.1.1.3 | Initial PET polymer hydrolysis |
|  | Putative hydrolase | mhqD | EC 3.1.- | General hydrolysis of polymer |
|  | Monoacylglycerol lipase  Putative esterase | -  - | EC 3.1.1.23  EC 3.1.-.- | BHET to MHET conversion |
|  | Carboxylesterase | CES | EC 3.1.1.1 | Initial PET polymer breakdown, hydrolyzes of ester bonds |
|  | Thermostable monoacylglycerol lipase | GMGL | EC 3.1.1.23 | Further breakdown of PET oligomers |
| Ring Structure Processing | | | | |
|  | Biphenyl dioxygenase alpha subunit | bphE | EC 1.14.12.18 | Initial ring structure attack |
|  | 3,4-dihydroxyphenylacetate 2,3-dioxygenase | hpcB | EC 1.13.11.15 | Aromatic ring cleavage |
|  | Gentisate 1,2-dioxygenase | sdgD2 | EC 1.13.11.4 | Ring opening of gentisate intermediates |
|  | Catechol 2,3-dioxygenase | catE | EC 1.13.11.2 | Catechol ring cleavage |
| Lactone and Carboxylic Acid Processing | | | | |
|  | 4-carboxymuconolactone decarboxylase | pcaC | EC 4.1.1.44 | Lactone processing |
|  | 4,5-dihydroxyphthalate decarboxylase | pht5 | EC 4.1.1.55 | Carboxylic acid modification |
|  | Gluconolactonase | pgl | EC 3.1.1.31 | Lactone hydrolysis |
| Alcohol and Aldehyde Processing | | | | |
|  | Alcohol dehydrogenase | adh2 | EC 1.1.1.1 | Alcohol group oxidation |
|  | Long-chain-alcohol dehydrogenase | adh1 | EC 1.1.1.192 | Complex alcohol oxidation |
|  | Short-chain dehydrogenase/reductase SDR | fabG | EC 1.1.1.100 | General oxidation of alcohols to aldehydes |
|  | Aldehyde dehydrogenase | adh1 | EC 1.2.1.3 | Aldehyde oxidation |
|  | Acetaldehyde dehydrogenase | bphJ | EC 1.2.1.10 | Simple aldehyde processing |
